# Efficient and versatile supramolecular functionalization of polysilicon microchips with cationic amphiphile salts for incorporation of therapeutics

**DOI:** 10.1007/s00604-026-08265-3

**Published:** 2026-07-16

**Authors:** María Elisa Alea-Reyes, Saman Bagherpour, Marta Duch, José Antonio Plaza, Lluïsa Pérez-García

**Affiliations:** 1 Departament de Farmacologia, Toxicologia i Química Terapèutica, Universitat de Barcelona, Avda. Joan XXIII 27-31, Barcelona, 08028 Spain; 2 Institut de Nanociència i Nanotecnologia UB (IN2UB), Universitat de Barcelona, Barcelona, 08028 Spain; 3 Institute of Microelectronics of Barcelona IMB-CNM (CSIC), Campus UAB, Cerdanyola, Barcelona 08193 Spain

**Keywords:** Polysilicon microchips, Non-covalent and covalent functionalization, Supramolecular functionalization, Gemini amphiphile salts, Fluorescence characterization

## Abstract

A major challenge in working with suspended microchips for biosensing and drug-delivery lies in selecting an approach that produces highly ordered self-assembled monolayers during functionalization, along with identifying straightforward techniques for their characterization and quantification. In the current work, we aim to overcome these challenges by employing different supramolecular functionalization methods to identify and optimize robust approaches for achieving homogeneous functionalization, particularly with microchips in suspension. For this purpose, polysilicon microchips (**PSµCs**), fabricated by photolithographic techniques, were functionalized with gemini pyridinium and imidazolium derivatives through either covalent or non-covalent approaches followed by supramolecular functionalization of a porphyrin, which was used as a fluorophore to enable characterization. To explore the versatility of the methodology, we alternatively functionalized **PSµCs** with gemini bipyridinium salts as encapsulating agents which incorporated π-excessive neurotransmitters in a supramolecular manner. Contact angle measurements and MALDI-ToF mass spectrometry confirmed the extent of surface functionalization. Fluorescence microscopy also verified the incorporation of porphyrin and neurotransmitters with high homogeneity on the released microchips in suspension. UV–vis absorption spectroscopy exhibited negligible release of the porphyrin, along with a high loading capacity. *In vitro* assays also showed high cell viability in the presence of functionalized microchips. Overall, this functionalization approach overcomes modification challenges by effective and versatile incorporation of therapeutic agents on patterned wafers or suspended microchips for the development of biomedical devices.

**Graphical Abstract:**

**Figure:**
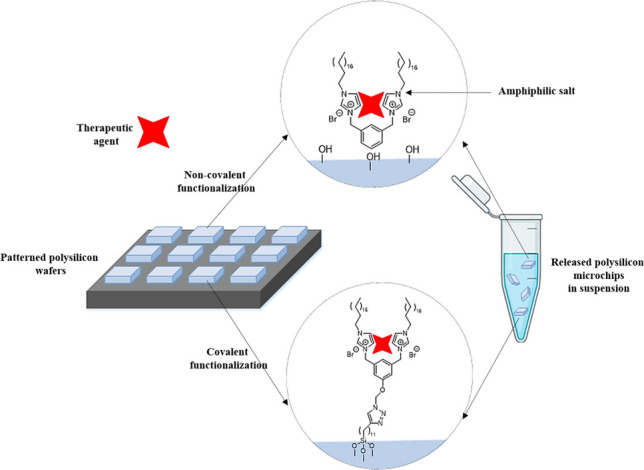

**Supplementary Information:**

The online version contains supplementary material available at 10.1007/s00604-026-08265-3.

## Introduction

The integration of micro- and nanotechnology has been recognized as highly significant in the field of biomedical science and its applications [1, 2]. Progress in micro- and nano-fabrication techniques has opened up new avenues for creating micro- and nano-devices, including proteins, DNA microarrays, and microfluidic circuits, that find utility in drug and gene delivery, tissue engineering, biosensing, as well as diagnostic systems [3–5].

Surface functionalization is a key strategy for imparting chemical and biological functionality to inert materials. Micro- and nanomaterials, for example microchips, can acquire physicochemical properties, such as controlled interactions with biomolecules, selective binding capabilities, and improved compatibility with biological environments, by introducing customized molecular groups or supramolecular assemblies at the surface [6]. However, achieving effective functionalization becomes considerably more challenging when microchips are processed in suspension rather than on planar substrates. Uniform surface modification and reproducibility in such systems may be hindered by restricted control over microchip orientation, decreased surface accessibility, and aggregation tendency [7–9]. Therefore, these challenges highlight the need for adaptable functionalization approaches capable of operating efficiently under suspension conditions.

Self-assembled monolayers (SAMs) have been widely employed as an effective strategy for the surface functionalization of a variety of nano- and microdevices [10–12]. Regarding functionalization in the suspension, one of the key challenges in the use of SAMs is to select a methodology that yields well-organized monolayers, as well as finding a simple method for characterization and quantification of the monolayer [13]. Moreover, it is crucial to establish a clear correlation between the selection of reagents capable of immobilization or supramolecular incorporation of active molecules, such as photosensitizers, on the surface of microchips, the associated chemical synthesis and characterization processes, and the resulting function of the newly developed material [14].

Gemini amphiphile salts have captured considerable attention due to their remarkable potential for several applications in various fields. For instance, gemini imidazolium amphiphiles have been studied for stabilization of gold nanoparticles [15], supramolecular gels [16], drug deliver and photodynamic therapy [17]. Moreover, bipyridinium-based compounds have shown their remarkable capabilities including the development of electrochromic displays, advancements in molecular electronics, and the creation of redox sensors [18]. This fascination arises from the intriguing interactions between π-electron-deficient bipyridinium segments and π-electron-rich donor compounds, which results in a well-defined host–guest interaction [19]. On the other hand, gemini amphiphiles offer several advantages over conventional surfactants due to their unique molecular architecture, which promotes enhanced self-assembly behaviour, lower critical micelle concentrations, and greater stability of the resulting supramolecular assemblies. These properties translated into a high capacity for molecular immobilization and surface organization, demonstrating the versatility of gemini amphiphiles for biomedical applications [20, 21]. Furthermore, their relatively low toxicity and favourable biocompatibility make them attractive candidates for pharmaceutical and surface-engineering applications, while their straightforward and high-yield synthetic route facilitates scalable and reproducible production [22, 23]. Therefore, these two types of amphiphilic salts can be promising candidates for the supramolecular functionalization and immobilization of active molecules on the surface of microchips in suspension.

Microfabrication techniques, such as photolithography, have been employed to create polysilicon microchips (**PSµCs**), which have demonstrated biocompatibility and found application in drug delivery [24]. Micrometric polysilicon microchips offer several advantages for intracellular applications compared to conventional organic carriers. Due to their rigid and well-defined micro-scale architecture, these microchips exhibit low polydispersity, minimizing aggregation phenomena commonly observed in organic nanomaterials within biological environments [25]. They also enable efficient cellular internalization and can be retained inside cells for extended periods, supporting long-term monitoring. In addition, polysilicon microchips are highly compatible with optical imaging techniques, as their controlled size and shape facilitate real-time tracking and quantitative analysis inside cells [26]. These combined features make them particularly attractive platforms for the functionalization, immobilization, and delivery of therapeutic agents, as well as for sensing applications. To achieve exceptional uniformity, our group has utilized microelectronic-based methods to produce micrometric-sized particles of diverse materials, shapes, and dimensions, outperforming chemically synthesized **PSµCs** in terms of polydispersity [25]. These **PSµCs** can effectively interact with living cells [27], serving as intracellular pH sensors [26], acting as micrometric tags for real-time cell tracking [28, 29], intracellular glutathione sensing [30], among other potential uses. However, one of the major challenges was to find a robust approach for the functionalization of these types of microchips, especially in suspension [31].

**Fig. 1:**
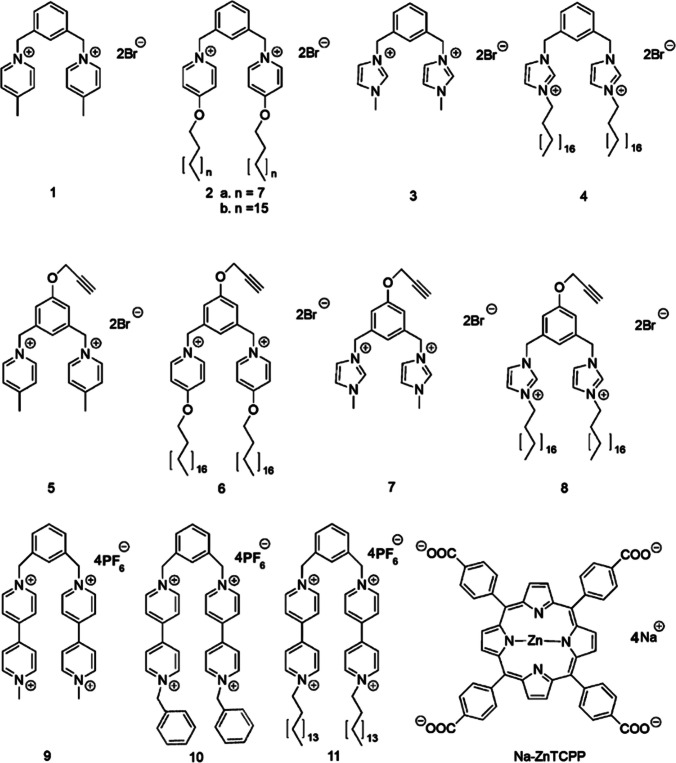
Molecular structures of the compounds involved in the functionalization of silicon microchips: gemini pyridinium and imidazolium salts **1**–**4** used in the non-covalent functionalization, gemini pyridinium and imidazolium salts **5**–**8** used in the covalent functionalization of the polysilicon substrates (**PWs**, **PWµCs**, and **PSµCs**), gemini bipyridinium salts **9**–**11** and the anionic porphyrin **Na-ZnTCPP**

**Fig. 2:**
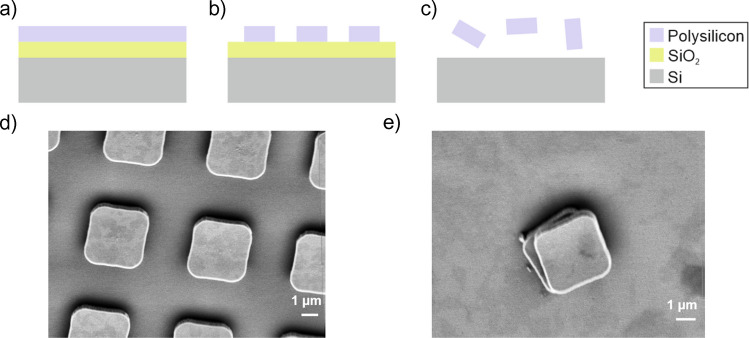
**a**–**c** Schematics of the fabrication process. **a** A 0.5 μm-thick polysilicon layer is deposited on a previously grown 1 μm-thick silicon oxide layer on a silicon wafer, forming the **PWs** substrates. **b** Photolithography followed by dry etching patterns the polysilicon layer into 3 × 3 μm^2^ microchips (3 μm pitch), yielding the PWμCs substrates. **c** The underlying silicon oxide layer is removed to release the chips from the wafer, resulting in suspended PSμCs. SEM images of **d** the structured microchips on the wafer and **e** after release

In this study, we aimed to develop and optimize a functionalization method using gemini amphiphilic salts as supramolecular immobilizing agents (Fig. 1) for functionalization of polysilicon substrates, including polysilicon-coated wafers (**PWs**) and microfabricated polysilicon wafers with structured microchips (**PWµCs)**, to later apply this approach for modification of polysilicon suspended microchips **(PSµCs)** (Fig. 2). Rather than presenting an analytical sensing or delivery application, the study establishes a robust functionalization platform as the central contribution, serving as a foundation for future application-driven developments. For this purpose, gemini pyridinium and imidazolium salts were synthesized and immobilized on the surface of **PWs** and **PWµCs** through both non-covalent and covalent approaches, followed by supramolecular immobilization of the metalloporphyrin zinc(II) meso-tetrakis(4-carboxyphenyl)porphyrin sodium salt (**Na-ZnTCPP**). The versatility of this approach was also evaluated by functionalizing these substrates with bipyridinium salts as encapsulating agents and incorporating the neurotransmitters dopamine (Dop), serotonin (Ser), adrenaline (Adr), and noradrenaline (Nor). Successful surface modification of **PWs** was evaluated by contact-angle measurements, and **PWµCs** supramolecular functionalization were characterized by fluorescence microscopy and MALDI-ToF mass spectrometry. The optimized functionalization approach was employed for supramolecular functionalization of **PSµCs**, and fluorescence microscopy served to confirm the presence of both porphyrins and neurotransmitters. Moreover, the loading capacity of the photosensitizers and neurotransmitters, as well as the cell viability of **PSµCs,** were also studied. Compared with our previously reported polysilicon microchip systems functionalized directly through silane coupling chemistry, the present approach provides a substantially more homogeneous surface modification. In our earlier studies, confocal microscopy revealed a less uniform distribution of immobilized fluorophores when functionalization was performed in suspension [27]. In contrast, the incorporation of gemini amphiphiles results in a more uniform and well-organized functional layer across the microchip surface, leading to improved surface coverage and reproducibility. This enhanced homogeneity is attributed to the strong self-assembly capability of the gemini amphiphiles, which promotes a more controlled and consistent surface functionalization process. Another key innovation of this work is the development of a supramolecular immobilization strategy for loading functional molecules onto polysilicon microchips without requiring chemical modification of the cargo. By relying on non-covalent interactions, this approach preserves molecular functionality and enables the immobilization of a broader range of compounds, including porphyrin photosensitizers and neurotransmitters.

## Experimental

Materials, general methods, and synthesis of compounds **1,2** and **5–7** are included in Supplementary Information.

### Functionalization of PWs or PWµCs with gemini cationic salts

#### Non-covalent functionalization of PWs or PWµCs with pyridinium (1) and (2), imidazolium salts (3) and (4), and bis-bipyridinium salts 9–11

The hydroxylated **PWs** (or **PWµCs**) were immersed in a solution of compound **1** (1.8 mg, 2 mM) or **3** (1.7 mg, 2 mM) in 2 mL of water. Alternatively, they were immersed in a solution of compound **2a** (2.9 mg, 2 mM), **2b** (3.8 mg, 2 mM), or **4** (3.6 mg, 2 mM) in chloroform. In each case, the mixture was stirred on an orbital shaker at 90 rpm for 6 h. Upon completion of the deposition time, the substrates were thoroughly rinsed with water (5 × 3 mL) in the case of wafers immobilized with compound **1** or **3**, and with chloroform (5 × 3 mL) in the case of **2** or **4**, and subsequently dried using nitrogen. In the case of bis-bipyridinium salts **9–11**, the activated **PWs** were immersed in a solution of either **9** (2.1 mg, 1 mM), **10** (2.4 mg, 1 mM) or **11** (2.9 mg, 1 mM) in DMSO (2 mL) and were stirred on a shaker at 90 rpm for 6 h followed by rinsing with DMSO (5 × 3 mL) and water (5 × 3 mL) and drying with nitrogen.

#### Covalent functionalization of PWs (or PWµCs) with pyridinium (5) and (6) and imidazolium salts (7) and (8)

The hydroxylated **PWs** (or **PWµCs**) were immersed in a solution of 11-azidoundecyltrimethoxysilane (1.3 mg, 2 mM) in toluene (2 mL). The **PWs** were stirred on an orbital shaker at 90 rpm for 24 h. Following this period, the wafers were washed with toluene (5 × 3 mL) and EtOH (3 × 3 mL) and dried with nitrogen. For the copper-catalyzed Huisgen cycloaddition, the silane-treated wafers were functionalized in a mixture containing copper sulphate (0.15 mg, 0.3 mM) in water (0.5 mL), sodium-L-ascorbate (0.40 mg, 1.0 mM) in water (0.5 mL), and compounds **5** (1 mg, 2 mM) in water (1 mL), **6** (2 mg, 2 mM) in DMSO (1 mL), **7** (0.96 mg, 2 mM) in water (1 mL), or **8** (1.9 mg, 2 mM) in DMSO (1 mL). These mixtures were stirred on an orbital shaker at 90 rpm for 24 h. After the deposition time, the substrates were rinsed thoroughly with water in the case of **PWs** immobilized with **5** and **7**, and with DMSO for compounds **6** and **8** (5 × 3 mL) and then dried by nitrogen.

### Functionalization of polysilicon microchips (PSµCs) with gemini cationic salts in suspension

#### Non-covalent functionalization of PSµCs with pyridinium 2b, imidazolium salts 4, and bis-bipyridinium salt 9

1 mL solution of the pyridinium salt **2b** (2 mM) or imidazolium salt **4** (2 mM) in CHCl_3_ were added to the microtubes containing surface activated **PSµCs**. All microtubes containing **PSµCs** were stirred on the continuous shaking at 400 rpm for 6 h. Then, the suspensions were centrifuged for 15 min at 13500 rpm and washed with their corresponding solvent used in the functionalization (5 × 1 mL) to eliminate the excess of the compound **2b** or **4**. Afterwards, the supernatant was removed and a solution of MTMS (0.4 mg, 1 mM) in water (1 mL) was added to the **PSµCs** and incubated for 2 h at 400 rpm. Afterwards, the suspensions were centrifuged for 15 min at 13500 rpm and washed with distilled water (5 × 1 mL) to eliminate the excess of MTMS and then air dried.

In the case of compound **9**, a solution of the bis-bipyridinium salt **9** (1.0 mg, 1 mM) in DMSO (1 mL) was added to the microtube containing surface activated **PSµCs** and stirred at 400 rpm for 6 h. Suspension was then centrifuged for 15 min at 13500 rpm and washed with DMSO (5 × 1 mL) to eliminate the excess of **9**.

#### Covalent functionalization of PSµCs with pyridinium 6 and imidazolium salts 8

A solution of 11-azidoundecyltrimethoxysilane (0.3 mg, 2 mM) in toluene (0.5 mL) was added to the substrates. All microtubes containing surface activated **PSµCs** were stirred at 400 rpm for 24 h. Afterwards, the suspensions were centrifuged for 15 min at 13500 rpm, washed with toluene (5 × 1 mL) and EtOH (3 × 1 mL), and dried under air flow. A solution of copper sulphate (0.04 mg, 0.3 mM) in water (123 μL), sodium-L-ascorbate (0.09 mg, 1.0 mM) in water (114 μL) and the compounds of **6** (0.5 mg, 2 mM) or **8** (0.5 mg, 2 mM) in DMSO (263 μL) were added to the microtubes. All the microtubes containing **PSµCs** were stirred with a shaker at 400 rpm for 24 h. Then, the **PSµCs** functionalized were centrifuged for 15 min at 13500 rpm, washed with the mixture H2O/DMSO (1:1) (5 × 1 mL), and dried under air flow. Finally, a solution of MTMS (0.4 mg, 1 mM) in water (1 mL) was added to the **PSµCs** and incubated for 2 h at 400 rpm. Then, the suspensions were centrifuged for 15 min at 13500 rpm and washed with distilled water (5 × 1 mL) to eliminate the excess of MTMS and then air dried.

### Immobilization of porphyrin and neurotransmitters into functionalized PWs, PWµCs, and PSµCs

#### Immobilization of the porphyrin Na-ZnTCPP into functionalized PWs, PWµCs, and PSµCs

The **PWs** (or **PWµCs**) functionalized with pyridinium salts 1, 2, 5, or 6, and imidazolium salts 3, 4, 7, or 8, were immersed in a solution of **Na-ZnTCPP** (3.7 mg, 2 mM) in 2 mL of water. They were stirred on an orbital shaker at 90 rpm for 24 h. Subsequently, the substrates were thoroughly rinsed with water (5 × 3 mL) to remove any excess **Na-ZnTCPP** and dried using nitrogen.

Regarding functionalized **PSµCs**, a solution of porphyrin **Na-ZnTCPP** (1.9 mg, 2 mM) in water (1 mL) was added to the covalent and non-covalent functionalized **PSµCs** with pyridinium salt **2b** or **6**, imidazolium salt **4** or **8** and MTMS. All microtubes were stirred by a shaker (400 rpm) for 48 h at room temperature. **PSµCs** were then centrifuged for 15 min at 13500 rpm, washed with water (5 × 1 mL) to eliminate the excess of porphyrin **Na-ZnTCPP** and dried with air flow.

#### Immobilization of neurotransmitters Dop, Ser, Adr, and Nor on PWs, PWµCs, and PSµCs

The functionalized **PWs** (or **PWµCs**) with each of **9–11** were immersed in a solution of either Dop (0.8 mg, 2 mM), Ser (0.9 mg, 2 mM), Adr (0.9 mg, 2 mM) or Nor (0.8 mg, 2 mM) in water (2 mL) and were stirred on an orbital shaker at 90 rpm for 24 h. After this time, the **PWs** were rinsed with distilled water (5 × 3 mL), to eliminate the excess of neurotransmitters and dried with nitrogen.

In the case of functionalized **PSµCs**, a solution of either Dop (0.4 mg, 2 mM) or Ser (0.5 mg, 2 mM) in H_2_O (1 mL) was added to the suspension of functionalized **PSµCs** with **9**. All microtubes were stirred for 24 h at room temperature at 400 rpm, and the suspensions were then centrifuged for 15 min at 13500 rpm, washed with H_2_O (5 × 1 mL) to eliminate the excess of Dop or Ser, and then the pellet was dried with nitrogen.

### Biological experiments

#### Cell culture medium

Imaging medium based on Hank’s balanced salt solution (HBSS) for the biological experiments was prepared in water containing NaCl (120 mM), KCl (5 mM), CaCl_2_∙2H_2_O (2 mM), MgCl_2_∙6H_2_O (1 mM), NaH_2_PO_4_ (1 mM), NaHCO_3_ (1 mM), HEPES (25 mM) and D-glucose (11 mM) and BSA (1 mg/mL). The pH of the imaging medium was adjusted to 7.4 using an aqueous solution of NaOH (1 M). The imaging medium was sterilized by filtration through a Millex GP syringe driven filter unit (0.22 µm) prior to use.

#### Phosphate buffer saline (PBS)

The PBS solution used for the biological experiments was prepared by dissolving 10 PBS tablets in water (1 L). The solution was sterilized by autoclaving at 110 °C for 10 min. The newly prepared PBS solution contained Na_2_HPO_4_ (8 mM), KH_2_PO_4_ (1 mM), NaCl (160 mM) and KCl (3 mM) with a pH value of 7.3.

#### MTT cell proliferation assay

HeLa cells (human cervical adenocarcinoma cells; ATCC CCL-2) were used to test the cell viability of the porphyrin **Na-ZnTCPP**, and the control of **PSµCs** and loaded porphyrins **Na-ZnTCPP-PSµCs**. HeLa cells were cultured at 37°C in a humidified sterile atmosphere of 95% air and 5% CO_2_, using DMEM supplemented with FBS (10% v/v), glucose (4.5 g L^−1^), L-glutamine (292 mg L^−1^), streptomycin sulphate (50 mg L^−1^) and potassium penicillin (50000 U L^−1^). For cell viability studies, cells were seeded in 96-well plates (3300 cells well^−1^) and grown up to 70–85% confluence. Cells were then incubated for 24 h with different concentrations of the free porphyrin (0–100 μM), the control **PSµCs**: (0–400000 µP/mL) or the **PSµCs** functionalized with porphyrin (0–400000 µP/mL). Cells incubated with the complete medium in the absence of **PSµCs** were also used as control. After incubation, the medium was discarded, cells were washed three times with cold PBS and incubation was followed for 24 additional hours with fresh culture medium. Cell viability was determined by means of the MTT assay. Briefly, the remaining HeLa cells were incubated with a MTT solution (0.05 mg mL^−1^) in complete DMEM for 3 h. The medium was discarded, and formazan crystals were solubilized with pure DMSO followed by determination of its concentration by absorption spectroscopy at 526 nm. Cell viability was determined by the ratio between the absorbance of treated cells and that of non-treated cells (control, 100% viability).

## Results and discussion

### Synthesis and characterization of cationic gemini amphiphiles

This work is fundamentally focused on materials engineering aspects, specifically achieving controlled and reproducible immobilization of functional molecules on the microchip surface. For this purpose, a group of cationic gemini amphiphiles comprising two cationic heterocyclic moieties, connected by a m-xylylene, each carrying hydrocarbon chains of varying number of carbons were selected (Fig. 1). The purpose of the selection was to investigate the impact of different structural modifications on surface functionalization and supramolecular immobilization processes of therapeutic molecules. The synthesized cationic gemini amphiphiles possess cationic moieties such as pyridinium (**1, 2, 5, 6**), imidazolium (**3, 4, 7, 8**), and bipyridinium (**9, 10, 11**). The heterocyclic units incorporate either short (methyl group) or long (10 or 18 carbon atoms) hydrocarbon chains. For compounds **5–8**, a propargyloxy group is present in the aromatic spacer, allowing conjugation to the polysilicon substrates **(PWs**, **PWµCs**, and **PSµCs**) covalently, while the rest of the compounds will undergo non-covalent functionalization via chemical adsorption. Compounds **1,2** and **5**–**7** were synthesized and characterized as described in Scheme S1, Section S1-1 to S1-3, and Figure S1-S31 in the Supplementary Information, whereas the synthesis of compounds **3**, **4, 8–11,** and **Na-ZnTCPP** has been reported elsewhere [20, 32–34]. **Na-ZnTCPP** offers several advantages, including water solubility and its anionic nature, which promotes favorable electrostatic interactions between its carboxylate groups and the positive charges of the pyridinium and imidazolium salts **1–8**, as well as some hydrogen bonding. The amphiphilic bipyridinium salts served as hosts for the non-covalent functionalization of neurotransmitters to the **PWs**, **PWµCs** and **PSµCs**.

### Fabrication of PWµCs and PSµCs

The fabrication of the three different samples was carried out starting from a p-type silicon wafer, Fig. 2. Initially, a 1 µm-thick layer of thermal silicon oxide was grown on the silicon wafers. Subsequently, a 500 nm-thick polysilicon layer was deposited using low-pressure chemical vapor deposition (LPCVD). At this stage, polysilicon-coated wafers (**PWs**) were obtained.

A photolithographic process was then performed to define microchips with lateral dimensions of 3 × 3 µm^2^. The devices were subsequently patterned by dry etching, followed by the removal of the photoresist. This process yielded polysilicon wafers with structured µChip (PWµC).

Finally, in order to release the polysilicon microchips and obtain suspended chips (**PSµCs**), the underlying 1 µm silicon oxide layer was selectively etched using hydrofluoric acid (HF) vapor. The released microchips were then collected in ethanol.

The initial focus was on functionalizing the wafers and adapting the surface modification methodology for microchip functionalization in suspension using compounds **1–8**, followed by immobilization of **Na-ZnTCPP**, or with compounds **9–11** and immobilization of neurotransmitters. Therefore, it is possible to find the optimized method for functionalization of microchips by characterizing the fluorescently labelled microchips in suspension.

### Non-covalent and covalent functionalization of polysilicon-coated wafers (PWs) and contact angle measurements

**Scheme 1:**
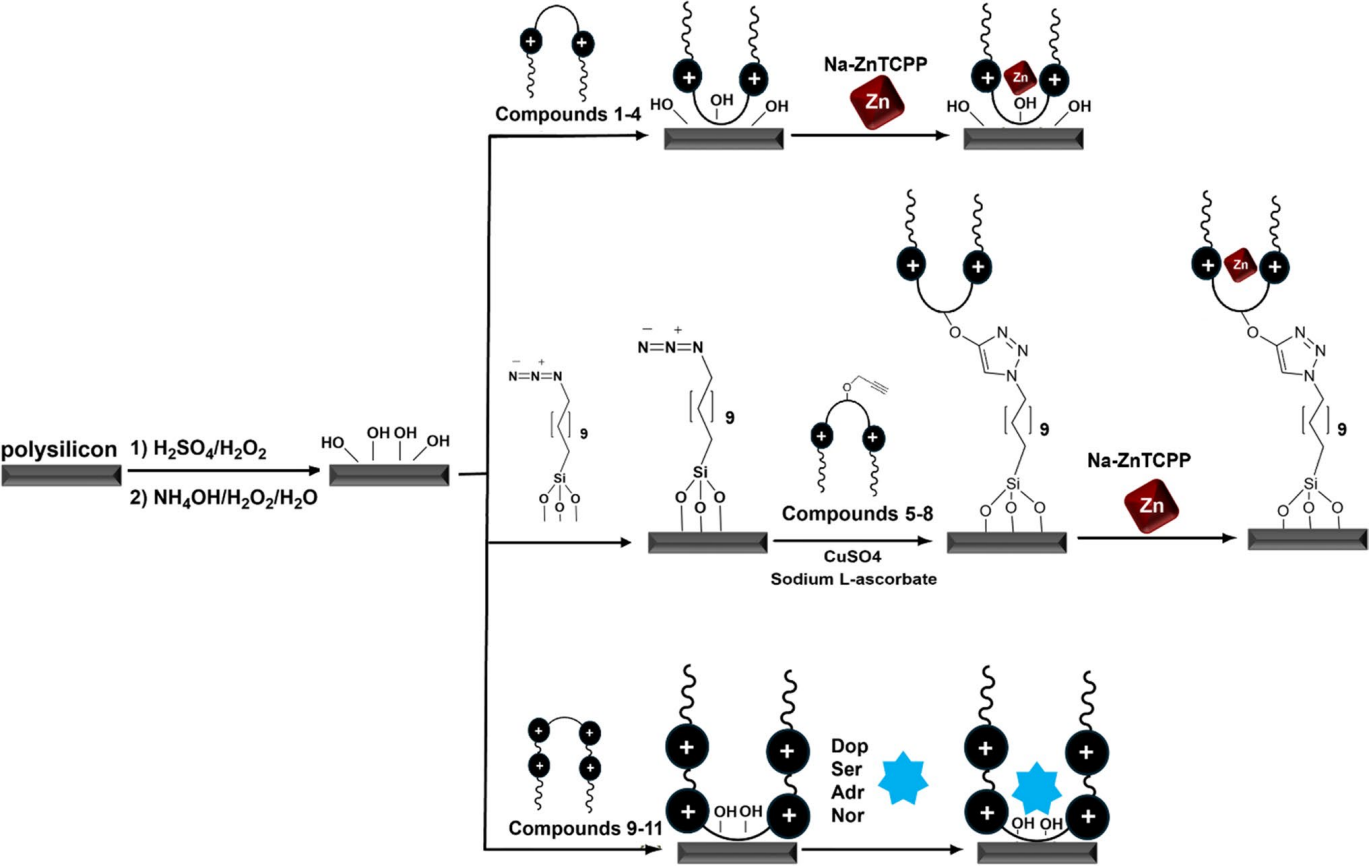
Non-covalent and covalent functionalization of the **PWs** with **1–4** and **5–8**, respectively, and immobilization of **Na-ZnTCPP** to the surface. Functionalization of **PWs** with bis- bipyridinium salts **9**, **10** or **11** and the subsequent incorporation of neurotransmitters Dop, Ser, Adr or Nor

The primary objective of this study is the development and optimization of a reliable surface functionalization strategy for polysilicon microchips using supramolecular chemistry. Two different approaches of non-covalent and covalent functionalization were carried out to immobilize compounds **1** to **11** on the **PWs** and **PWµCs** in order to perform the supramolecular incorporation of therapeutic substances. The non-covalent immobilization of the porphyrin **Na-ZnTCPP** into the **PWs** involved an optimized three-step process depicted in Scheme 1: a) activation using piranha solution H_2_SO_4_/H_2_O_2_ (7:3) followed by treating with a NH_4_OH/H_2_O_2_/H_2_O (1:1:5) solution; b) adsorption of compounds **1–4** by wet chemistry; c) the anionic porphyrin **Na-ZnTCPP** immobilization on each **1–4** monolayer (Scheme 1). On the other hand, to achieve the covalent immobilization of the wafers with compounds of **5–8**, a four-step process was followed as illustrated in Scheme 1: a) surface activation (as before); b) siloxane bearing an azide functional group; c) linking various compounds of **5**–**8** containing the alkyne group through click chemistry to the surface of microchips; d) supramolecular immobilization of **Na-ZnTCPP** to the **PWs**. For compounds of **9**–**11**, a non-covalent functionalization mentioned above was conducted, followed by the immobilization of different neurotransmitters such as Dop, Ser, Adr, and Nor.

Contact angle measurements were performed to characterize all different **PWs** after functionalization and the values are displayed in Table 1. The contact angle values for the **PWs** after washing and activation were 16 ± 2°. In all cases of **PWs** functionalized with the different compounds **1–8**, an increase in contact angle values was observed compared to the controls (**PW4-PW12** in Table 1). The highest contact angle values were obtained for **PWs** functionalized with pyridinium salts **2b** and **6**, and imidazolium salts **4** and **8**.

**Table 1:** Contact angles measurements of **PWs** functionalized with the compounds **1**–**8** and subsequent immobilization of porphyrin **Na-ZnTCPP**, **PWs** functionalized with **9**–**11**, and subsequent incorporation of neurotransmitters Dop, Ser, Adr and Nor

Surfaces (PWs)	Functionalization condition ^a^	ϴ^b^ (°) ± SD^c^(°)	Surfaces (PWs)	Functionalization condition ^a^	ϴ^b^ (°) ± SD^c^(°)
PW1	Control (H_2_O)	16 ± 1	**PW22**	Control (MeCN)	20 ± 1
PW2	Control (CHC_3_)	23 ± 1	**PW23**	9	57 ± 1
PW3	Na-ZnTCPP	20 ± 2	**PW24**	10	66 ± 1
PW4	1	38 ± 1	**PW25**	11	89 ± 1
PW5	2a	53 ± 2	**PW26**	Dop	27 ± 2
PW6	2b	83 ± 1	**PW27**	Ser	25 ± 1
PW7	3	32 ± 1	**PW28**	Adr	23 ± 2
PW8	4	77 ± 1	**PW29**	Nor	21 ± 1
PW9	5	50 ± 1	**PW30**	9 + Dop	48 ± 1
PW10	6	94 ± 1	**PW31**	9 + Ser	44 ± 1
PW11	7	45 ± 1	**PW32**	9 + Adr	40 ± 1
PW12	8	88 ± 1	**PW33**	9 + Nor	43 ± 1
PW13	1 + Na-ZnTCPP^c^	32 ± 1	**PW34**	10 + Dop	57 ± 1
PW14	2a + Na-ZnTCPP^c^	48 ± 2	**PW35**	10 + Ser	55 ± 1
PW15	2b + Na-ZnTCPP	78 ± 1	**PW36**	10 + Adr	50 ± 2
PW16	3 + Na-ZnTCPP	28 ± 1	**PW37**	10 + Nor	52 ± 2
PW17	4 + Na-ZnTCPP	70 ± 2	**PW38**	11 + Dop	73 ± 2
PW18	5 + Na-ZnTCPP	42 ± 1	**PW39**	11 + Ser	69 ± 2
PW19	6 + Na-ZnTCPP	88 ± 2	**PW40**	11 + Adr	70 ± 1
PW20	7 + Na-ZnTCPP^c^	40 ± 1	**PW41**	11 + Nor	66 ± 1
PW21	8 + Na-ZnTCPP	80 ± 2			

^a^ Functionalization conditions refer to the functionalization of PWs with the corresponding controls (solvent) or compounds mentioned in the table. ^b^ Contact angles. ^c^ Standard deviation of the contact angle values

These results indicate successful functionalization of the **PWs**, where the outward-facing part of the molecule is the aliphatic moiety while the polar head is adsorbed onto the surface, making it more hydrophobic. Importantly, after treatment with porphyrin **Na-ZnTCPP**, the contact angle values notably decreased (**PW13-PW21** in Table 1), indicating the presence of the hydrophilic porphyrin **Na-ZnTCPP** in the **PWs**. Regarding compounds **9**–**11**, there is an increase in contact angle values, attributed to the hydrophobic nature of the bis-bipyridinium salts (**PW23-PW25** in Table 1).

Notably, the **PWs** functionalized with **11** exhibited a higher value (**PW25**), suggesting increased hydrophobicity due to the longer alkyl chain containing 18 carbon atoms. Following the incorporation of any of the four different water-soluble neurotransmitters onto the **PWs**, the contact angle values decreased significantly (**PW30-PW41** in Table 1), indicating the presence of water-soluble π-electron-rich neurotransmitters Dop, Ser, Adr, or Nor.

To confirm the presence of gemini amphiphiles on the **PWs**, three different samples, namely **PW4**, **PW5**, and **PW6** incorporated on **PWs**, were chosen for characterization using MALDI-ToF–MS. Previous studies have employed this technique to analyze self-assembled monolayers (SAMs) on various materials like gold [35] or polysilicon surfaces [36, 37]. In our case, the experiments to analyze the functionalized **PWs** using MALDI-ToF–MS were conducted. MALDI-TOF spectra of surface-functionalized samples analysis on polysilicon-based surfaces may lead to partial fragmentation and the formation of cluster and adduct ions rather than exclusively intact molecular ions. In addition, the use of DHB as a matrix can promote dimerization and cluster formation, while trace alkali metal ions (Na⁺/K⁺) commonly present in the system can generate multiple adduct species. These combined effects may result in variations in peak patterns and reduced one-to-one correspondence between solution-phase molecular weights and surface-measured MALDI spectra. In all cases, the corresponding peaks of the possible adduct-related species of pyridinium salts related to compound **1**, **2a**, and **2b** are shown in Figure S32-S34, confirming the successful functionalization of **PWs**.

### Characterization of functionalized polysilicon wafers (PWµCs) by fluorescence microscopy

**Fig. 3:**
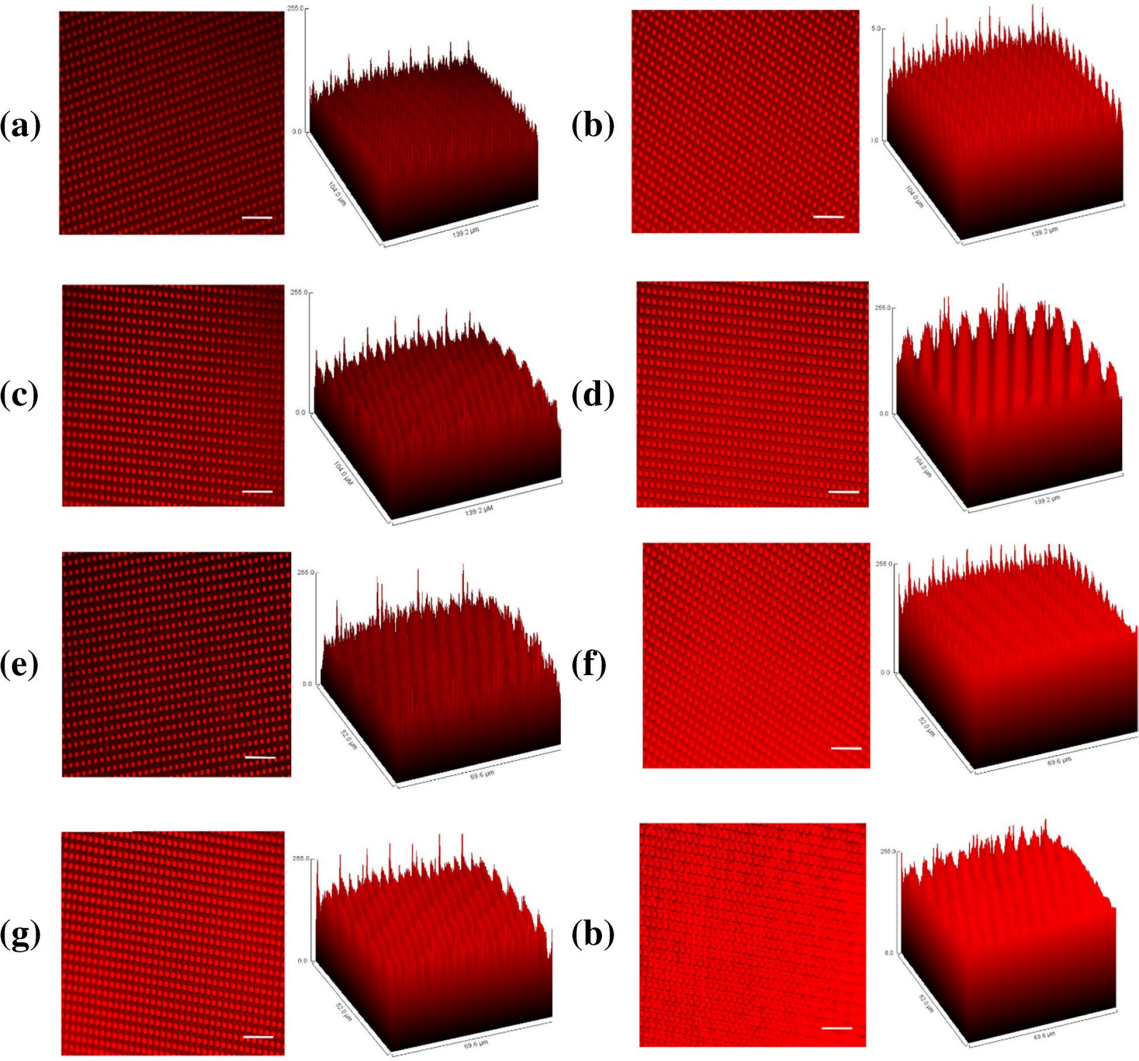
Fluorescence optical microscope images of functionalized **PWµCs** (having a pattern of 3 × 3 μm^2^ polysilicon microchips) with compound **a** **1**, **b** **2b**, **c** **3**, **d** **4**, **e** **5**, **f** **6**, **g** **7**, or **h** **8** and subsequent immobilization of **Na-ZnTCPP** with their corresponding 3D surface plots. Scale bars = 20 μm

**PWµCs** were functionalized using the same approach applied for functionalization of **PWs,** and fluorescence microscopy was utilized to confirm the incorporation of either **Na-ZnTCPP** or neurotransmitters. Figure 3 exhibits the different functionalized **PWµCs** and their 3D surface plots after supramolecular immobilization of **Na-ZnTCPP**. The samples were excited using a 552 nm laser diode, and the emitted fluorescence was detected between 600 and 700 nm. Before incorporation of **Na-ZnTCPP**, **PWµCs** do not exhibit fluorescent emission in both types of non-covalent (compounds **1**–**4**) and covalent (compounds **5**–**8**) functionalization. However, the **PWµCs** functionalized with compounds **1** to **8** and encapsulated by the anionic porphyrin **Na-ZnTCPP** displayed a significant increase in fluorescence intensity (Fig. 3), indicating successful immobilization of **Na-ZnTCPP** on the **PWµCs**. For **PWµCs** functionalized with compounds possessing longer aliphatic chains (**2b** (**2a** is shown in Figure S35 in Supplementary Information), **4**, **6**, and **8**) which are exhibited in Fig. 3b, d, f, h, respectively, a considerable enhancement in fluorescence intensity was observed in comparison to the less hydrophobic compounds of **1**, **3**, **5**, and **7** shown in the Fig. 3a, c, e, g, respectively. This difference may be attributed to the length of the aliphatic chains that facilitate the proximity of the porphyrin to the polar head. Furthermore, the results obtained from both types of functionalization (non-covalent and covalent) of **PWµCs** showed no significant differences in the fluorescence intensity of the immobilized porphyrin, as illustrated in Figure S36. These results demonstrate that both methods are suitable protocols for the non-covalent and covalent immobilization of pyridinium and imidazolium salts on **PWµCs**. The values in Figure S36 were calculated based on the highest fluorescence intensity readings achieved from 3D surface plots shown in Fig. 3, considering that a maximum fluorescence intensity corresponds to 255 pixels. These findings also confirm that the fluorescence intensity increases for **PWµCs** functionalized by the bis-pyridinium or imidazolium gemini amphiphiles having the longer carbon chains.

The release of **Na-ZnTCPP** molecules was analysed for **PWµCs** functionalized with compound **2, 4, 6, and 8** using UV–Vis absorption spectroscopy (Figure S37), showing released amounts of approximately 0.025% for compounds **2** and **4** and 0.080% for compounds **6** and **8** for more than 100 h. In both cases, the maximum released amount is negligible compared to the total incorporated porphyrin, indicating strong interactions between the carboxylate porphyrin **Na-ZnTCPP** and the host molecules in the **PWµCs**. This behaviour is consistent with strong non-covalent interactions between the amphiphilic salt and porphyrin, resulting in efficient retention of the cargo on the microchip surface. Such strong association is advantageous for the intended application, as it enables prolonged immobilization of the functional payload, particularly for poorly water-soluble therapeutic agents that are prone to aggregation. Importantly, this stable retention supports the preservation of the functional integrity of the loaded compounds and ensures sustained performance of the microchips in both therapeutic delivery and sensing applications.

**Fig. 4:**
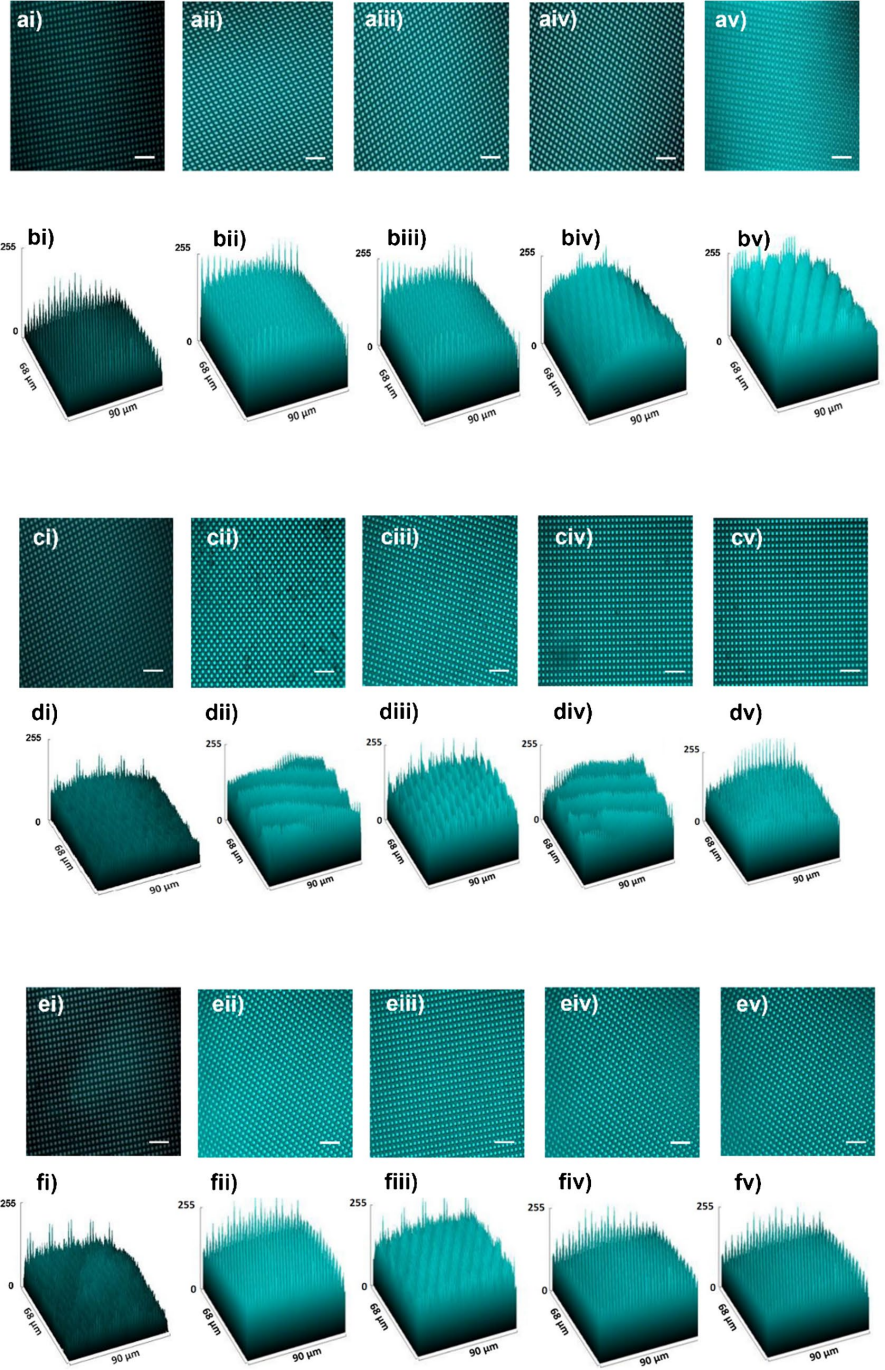
Fluorescence optical microscope image and 3D images of **PWµCs** with 3 × 3 µm^2^ polysilicon microchips: after functionalization only with ai-bi) **9**, ci-di) **10** and ei-fi) **11**, respectively. **PWµCs** functionalized with **9** and incorporation of the neurotransmitters aii-bii) Dop, aiii-biii) Ser, aiv-biv) Adr or av-bv) Nor, respectively. **PWµCs** functionalized with **10** and incorporation of the neurotransmitters cii-dii) Dop, ciii-diii) Ser, civ-div) Adr or cv-dv) Nor, respectively. **PWµCs** functionalized with **11** and subsequent incorporation of the neurotransmitters eii-fii) Dop, eiii-fiii) Ser, eiv-fiv) Adr or ev-fv) Nor, respectively. Scale bars = 20 μm

Figure 4 shows the fluorescence microscopy images of functionalized **PWµCs** with compound **9**–**11** (a filter of excitation in blue (BP 450–490) and emission (LP-515)) and after functionalization with Dop, Ser, Adr, or Nor. As it is observed in Fig. 4ai, ci, and ei, functionalized **PWµCs** with compound **9, 10, 11** exhibits a weak fluorescent emission in the excitation wavelength and emission range has been set for characterization of Dop, Ser, Adr, or Nor. This weak fluorescence intensity is also observed for the non-functionalized wafers which are shown in Figure S38 in Supplementary Information. A weak background signal was observed for the non-functionalized **PWµCs** under the detector gain settings used for fluorescence imaging. This signal is attributed to reflection/scattering from the polysilicon microstructures rather than to intrinsic fluorescence and does not correspond to compounds** 9**, **10**, or **11**.

The fluorescent intensity was significantly augmented after different neurotransmitters were encapsulated by compound 9 as shown in Fig. 4aii, cii, and eii, as well as the 3D surface plots (Fig. 4bii, dii, and fii). Figure 4 also shows the fluorescence microscopy images related to the functionalized PWµCs by compounds 10 and 11 indicating the increase in fluorescent intensity after immobilization π-electron-rich neurotransmitters. The control samples have also been prepared for non-functionalized PWµCs immersed in the neurotransmitters which can be seen in Figure S39.

As depicted in Figure S40, the PWµCs that underwent functionalization with bis-bipyridinium salt and subsequent immobilization of neurotransmitters exhibited fluorescence intensity values ranging from 35 to 65%. Conversely, **PWµCs** solely immersed in the neurotransmitter’s solutions displayed the fluorescence intensity values within a range of 15% to 21%. These findings potentially result from the electrostatic interaction formed between activated **PWµCs** and the electron-rich groups of neurotransmitters, resulting in a reduction of the inherent material fluorescence.

Following the functionalization process with compound **9** and supramolecular immobilization of neurotransmitters, the **PWµCs** exhibited elevated fluorescence intensity values within a range of 48% to 63%, as compared to compounds **10** and **11** with the same incorporated neurotransmitters (Figure S40). These outcomes can likely be attributed to the fact that compound **9** incorporates a smaller substituent in its structure, thereby facilitating interaction between the two pyridinium rings of compound **9** and the electron-rich groups of the neurotransmitters while maintaining an optimal distance for π-π interactions. Furthermore, the constrained rotation degree between the two pyridinium rings is likely responsible for the disparity in fluorescence intensity. Specifically, the heightened fluorescence intensity can be attributed to the complete restriction of pyridinium ring rotation within the complex of bis-bipyridinium salt and neurotransmitters.

It is worth noting that homogeneous functionalization of all microchip faces cannot be achieved while the microchips remain attached to the wafer. During wafer-based functionalization, the face in direct contact with the silicon oxide surface is not accessible to the functionalization solution and therefore remains unmodified. Furthermore, release of the microchips requires exposure to HF vapor, a harsh treatment that would compromise or destroy previously formed surface monolayers. Consequently, functionalization prior to release is not suitable when complete and intact surface modification is desired. To ensure uniform functionalization of all microchip surfaces while preserving the integrity of the molecular layer, the microchips must first be released from the wafer and subsequently functionalized in suspension.

A limitation of this study is that direct chemical characterization of the surface-bound layer could not be achieved. The intrinsic roughness of the polysilicon substrate prevented reliable AFM measurements, while FT-IR analysis was limited by the difficulty of preparing a sufficient number of released microchips for measurement. In addition, the surface coverage of the functional layer was below the detection limit of SEM–EDX relative to the bulk polysilicon substrate. Consequently, surface modification was inferred from contact-angle measurements and fluorescence microscopy, as well as MALDI-ToF–MS.

### Microchips functionalization in suspension and characterization

**Fig. 5:**
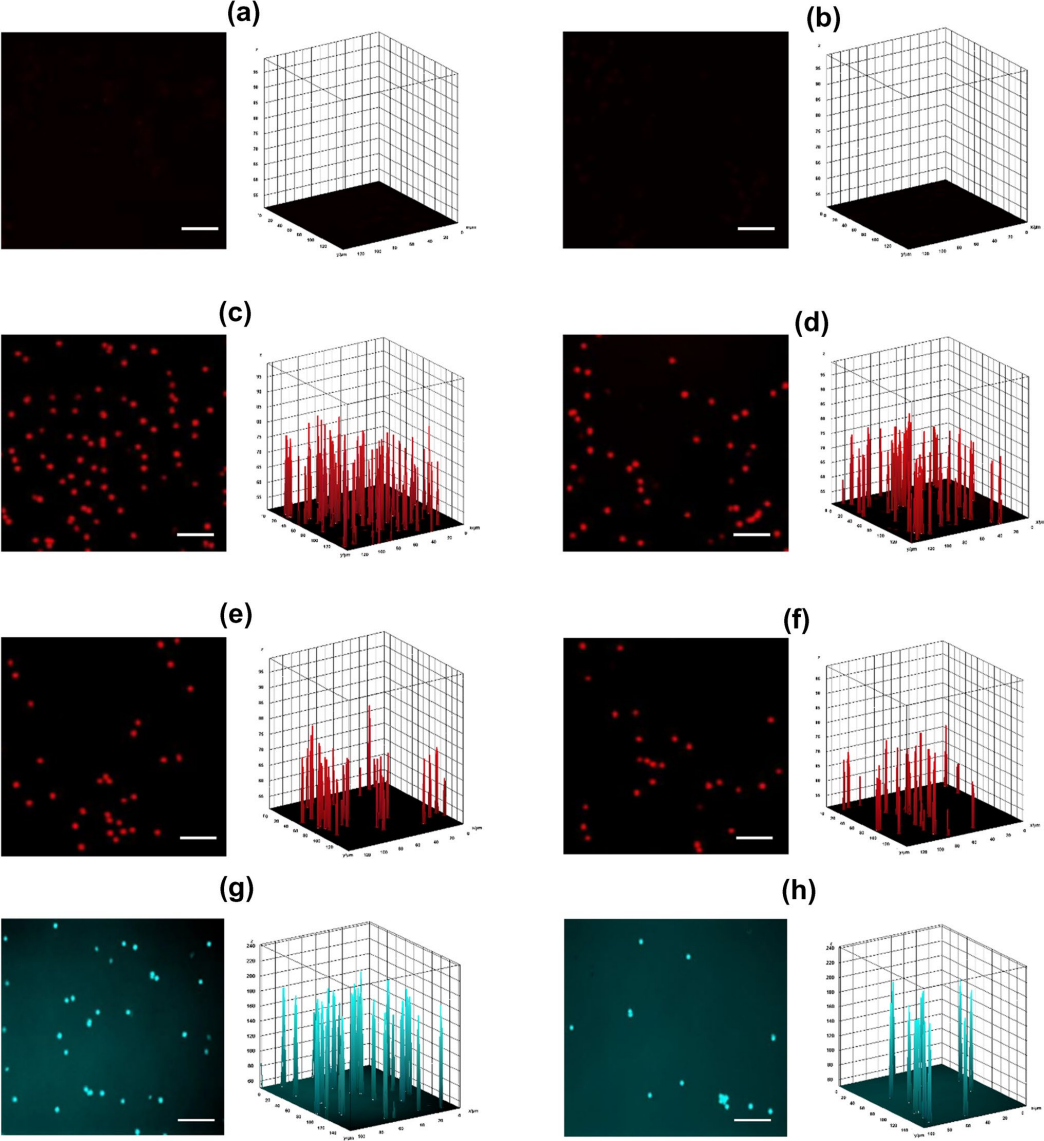
Fluorescence microscopy images of **PSµCs** and corresponding 3D images: **a** control (**PSµCs** non-functionalized) **b** after immersion only in **Na-ZnTCPP** solution, **c** and **d** non-covalent functionalization of **PSµCs** with the compounds **2a** or **4** and subsequent immobilization of **Na-ZnTCPP**, respectively, **e** and **f** covalent functionalization of **PSµCs** with **6** or **8** and subsequent immobilization of **Na-ZnTCPP**, respectively (These samples were excited using a 552 nm laser diode, and the emitted fluorescence was detected between 600 and 700 nm). Non-covalent functionalization of **PSµCs** with **9** and subsequent immobilization of: **g** the neurotransmitter Dop and **h** Ser. (These samples were excited by a filter of excitation in blue (BP 450–490) and emission (LP-515)) and their corresponding 3D fluorescence intensity projection. Scale bars = 20 μm

Immobilized compounds **2a**, **2b**, **4**, **6**, and **8** exhibited higher incorporation of **Na-ZnTCPP** and were chosen for supramolecular functionalization of polysilicon microchips in suspension (**PSµCs**). A mixed monolayer incorporating a siloxane (0.04% (w/v)) containing polyethylene glycol groups was used to enhance the water dispersibility and biocompatibility of the functionalized **PSµCs** in suspension. Fluorescence microscopy confirmed the immobilization of **Na-ZnTCPP** on the surface functionalized polysilicon microchips (**Na-ZnTCPP-PSµCs**), which is shown in Fig. 5. The quantification of immobilized **Na-ZnTCPP** was performed using UV–visible absorption spectroscopy. In the case of control **PSµCs** (Fig. 5a), **PSµCs** exclusively suspended in the **Na-ZnTCPP** solution (Fig. 5b), and **PSµCs** after surface activation and subsequent suspension in the **Na-ZnTCPP** solution (Figure S41), no fluorescence signal was detected. From these experiments, it can be concluded that the direct immobilization of **Na-ZnTCPP** on the surface of microchips without previous functionalization with a gemini amphiphilic salt is not possible. However, **PSµCs** functionalized with pyridinium salts **2a**, **2b** (Figure S41 in Supplementary Information), or **4** (or imidazolium salts **6** or **8**), followed by the incorporation of **Na-ZnTCPP**, exhibited a significant fluorescence, indicative of the presence of porphyrin **Na-ZnTCPP**.

Figure 5c shows the 3D fluorescent intensity plot for sample of **2a** which exhibits a significant difference compared to its control. Calibration curves spanning a range of 0.50–10 μM of porphyrin **Na-ZnTCPP** were employed for the porphyrin quantification (refer to Supplementary Information Figure S42). The results in Table S1 indicate that **Na-ZnTCPP-PSµCs** samples contained concentrations within the range of 2.2–2.6 μM of incorporated **Na-ZnTCPP**, and after the functionalization of the **PSµCs**, the number of **Na-ZnTCPP-PSµCs** per milliliter was assessed using a Neubauer chamber which was 800,000 PSµCs**/**mL, indicating the high efficiency of functionalization protocol in the suspension.

Compound **9** was also selected for the immobilization of two water soluble neurotransmitters of Dop and Ser on **PSµCs** using the protocol exhibited in Scheme 1. The presence of the encapsulated neurotransmitters Dop or Ser on the **PSµCs** was characterized by fluorescence microscopy (Fig. 5g and h, respectively), demonstrating a green fluorescence due to the formation of the complex bis-bipyridinium salt-neurotransmitters.

Under the fluorescence microscope and considering 255 pixels as the maximum intensity of fluorescence, the functionalized PSµCs with 9 and subsequent incorporation of Dop or Ser showed an average value for the fluorescence intensity of 71 and 72%, respectively, as shown in their 3D images (Fig. 5g and h, respectively). Non-functionalized PSµCs, functionalized only with compound 9, and suspended PSµCs in Dop or Ser solutions exhibited a low fluorescence (see Supplementary Information Figure S43), which is attributed to the auto-florescence of the polysilicon material in this emission range.

Additionally, the quantification of Dop and Ser incorporated in the previously functionalized **PSµCs** with compound **9** showed a concentration of 3.5 and 3.2 µM of Dop and Ser (Figure S44) which corresponds to the suspensions of 950000 PSµCs/mL.

These results demonstrate that the method used for the functionalization of **Na-ZnTCPP** and neurotransmitters can be applied not only to planar surfaces and patterned wafers, but also to microchips in suspension, overcoming the challenges associated with their functionalization while achieving high efficiency and a high yield of functionalized microchips during their preparation. Moreover, this study establishes clear structure–property relationships that can guide the design of future functionalized micro- and nanodevices. Longer alkyl chains promote more ordered interfacial monolayers, enhancing porphyrin proximity, fluorescence intensity, and host–guest interactions, while headgroup identity modulates electrostatic and π–π interactions with cargo molecules. While both methods are effective for stable and high-performance surface functionalization and fluorescence intensity, the non-covalent strategy offers a clear practical advantage in terms of synthetic simplicity, operational efficiency, and scalability, making it the more favourable route for polysilicon microchips functionalization.

### Cell viability assessments of Na-ZnTCPP-PSµCs

**Fig. 6:**
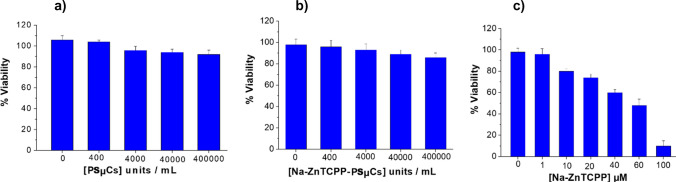
Cell viability data of the **a** non-functionalized **PSµCs** as control, **b Na-ZnTCPP**-**PSµCs**, **c** and free porphyrin **Na-ZnTCPP** using HeLa cell line. Results displayed as average with error bars corresponding to standard deviation, obtained for n = 3 independent experiments

To assess the suitability of **Na-ZnTCPP-PSµCs** for future phototoxicity studies, it is crucial to evaluate their potential cell viability *in vitro*. The non-functionalized **PSµCs**, **Na-ZnTCPP-PSµCs**, and free porphyrin **Na-ZnTCPP** in solution were used to treat HeLa cells, and the MTT cell proliferation assay was employed to assess the cell viability of the functionalized **Na-ZnTCPP-PSµCs** [38]. Interference of the silicon microchips with the MTT assay is considered negligible, as most non-internalized material is removed during washing. In addition, the porphyrin absorption does not overlap with the MTT formazan detection wavelength (λ = 526 nm), indicating no expected interference with absorbance measurements. In all cases, the viability of the control cells remained high, approximately 95% (Fig. 6a, b, and c). Similar results were obtained for the cells treated with non-functionalized **PSµCs** as a control (Fig. 6a) and **Na-ZnTCPP-PSµCs** (Fig. 6b), where the viability remained around 80% even at the highest evaluated concentration, as shown in Fig. 6a and b. However, the viability of HeLa cells treated with **Na-ZnTCPP** clearly decreased, as shown in Fig. 6c, and this decrease was consistent with the increase in the concentration of the photosensitizer. However, at the maximum concentration value studied for the incorporated porphyrin **Na-ZnTCPP** into **Na-ZnTCPP-PSµCs**, it was not cytotoxic for HeLa cells, with a cell viability of approximately 80% (Fig. 6c). Based on these results, the functionalized PSµCs prepared using the method established in this study can be considered low-cytotoxic microchips, as confirmed by preliminary cytocompatibility tests in HeLa cells.

## Conclusions

In this work, two approaches, non-covalent and covalent functionalization, were investigated using synthesized bis-pyridinium, bis-imidazolium, and bis-bipyridinium salts as host molecules for the supramolecular incorporation of the porphyrin **Na-ZnTCPP** or π-excessive neurotransmitters, with the aim of developing a robust and optimized method for the functionalization of polysilicon microchips **PSµCs** in suspension. To establish the protocol, first polysilicon-coated wafers (**PWs**) and patterned polysilicon wafers (**PWµCs**) were functionalized, and the optimized functionalization was confirmed by using contact angle measurements and MALDI-ToF–MS for **PWs**, as well as the fluorescence microscopy for **PWµCs**. The optimized functionalization method for **PSµCs** in the suspension showed the successful and homogeneous incorporation of non-covalent and covalent bis-pyridinium-**Na-ZnTCPP** and imidazolium-**Na-ZnTCPP** with the concentrations of 2.2–2.6 μM for suspensions containing 800,000 μP mL⁻^1^, indicating that both approaches provide similar efficiency for supramolecular immobilization of this photosensitizer. Only a negligible amount of incorporated porphyrin (~ 0.025%) was released, indicating stable interaction with the host systems. The applied method also demonstrated a homogeneous non-covalent functionalization of bis-bipyridinium-neurotransmitters on the **PSµCs** with the concentration of 3.5–3.2 μM corresponding to 950000 PSµCs/mL. Cell viability studies on HeLa cells showed that free **Na-ZnTCPP** reduced cell viability in a concentration-dependent manner, whereas **Na-ZnTCPP-PSµCs** and bare **PSµCs** maintained viability at around 80% at the highest microparticle concentration tested. The results of this study indicate that amphiphile structure, particularly longer alkyl chains (compounds 2b, 4, 6, and 8), has a greater influence on the fluorescence signal output than the immobilization approach. However, the non-covalent approach offers a clear practical advantage due to its simpler, catalyst-free, and less time-consuming implementation, making it more favourable for scalable and operationally efficient device fabrication. Overall, the incorporation of gemini amphiphiles enables a markedly more homogeneous and well-organized functionalization of polysilicon microchip surfaces compared with conventional silane-based approaches. This improved self-assembled architecture enhances surface coverage and reproducibility, addressing variability observed in earlier suspension-based methods. In addition, the supramolecular, non-covalent immobilization strategy allows efficient loading of diverse functional molecules without chemical modification, thereby preserving their native activity. Therefore, the functionalization approaches developed in this study can be applied not only for the immobilization or encapsulation of photosensitizers, biomolecules, and therapeutic agents on patterned wafers, but also for the functionalization of microchips in suspension. This approach overcomes the challenges associated with their modification and enables high functionalization efficiency during the preparation of microchips for biomedical devices.

## Supplementary Information

Below is the link to the electronic supplementary material.

Supplementary file1 (DOCX 12357 KB)

## Data Availability

The datasets generated during and/or analysed during the current study are available from the corresponding author on reasonable request.
