# Supplementary material for: Efficient and versatile supramolecular functionalization of polysilicon microchips with cationic amphiphile salts for incorporation of therapeutics
Source: Mikrochim Acta. 2026 Jul 16;193(8):549. doi: 10.1007/s00604-026-08265-3 (PMC13372932; doi:10.1007/s00604-026-08265-3)
Supplement: Supplementary file 1 — Supplementary file1 (DOCX 12357 KB) [file 604_2026_8265_MOESM1_ESM.docx]

**Supplementary Information**

**Versatile Supramolecular Functionalization of Polysilicon Microchips with Cationic Amphiphile Salts for Incorporation of Therapeutics**

María Elisa Alea-Reyes,^a,b^ Saman Bagherpour,^a,b^ Marta Duch^c^, José Antonio Plaza^c^, and Lluïsa Pérez-García*^a,b^

^a^ Departament de Farmacologia, Toxicologia i Química Terapèutica, Universitat de Barcelona, Avda. Joan XXIII 27-31, 08028 Barcelona, Spain. E-mail: mlperez@ub.edu

^b^ Institut de Nanociència i Nanotecnologia UB (IN2UB), Universitat de Barcelona, 08028 Barcelona, Spain.

^c^ Institute of Microelectronics of Barcelona (IMB-CNM, CSIC), Campus UAB, 08193, Cerdanyola, Barcelona, Spain.

**S1. Materials and Methods**

**S1.1 Materials**

***Solvents:*** Acetonitrile (MeCN), nitromethane, dichloromethane (DCM), methanol (MeOH), diethyl ether, isopropyl ether, chloroform (CHCl_3_), ethanol (EtOH), toluene, dimethyl sulfoxide (DMSO), tetrahydrofuran (THF), dimethylformamide (DMF), dimethylsulfoxide-d6 (CD_3_)_2_SO, chloroform-d (CDCl_3_) and deuterium oxide (D_2_O) were purchased from Sigma-Aldrich (Germany).

***Commercial compounds:*** 4-Methylpyridine, 1,3-bis(bromomethyl)benzene, 1-decanol, 1-octadecanol, 4-chloropyridine hydrochloride, hydrogen peroxide (30%), 1-methyl imidazole, ammonium hydroxide (20%), copper sulphate pentahydrate, sodium L-ascorbate, 11-azidoundecyltrimethoxy silane, anthracenediyl-bis(methylene)dimalonic acid (ABMA) 4,4′,4′′,4′′′-(Porphine-5,10,15,20-tetrayl)tetrakis(benzoic acid) (H2TCPP), zinc acetate, 4,4'-bipyridine, iodomethane, ammonium hexafluorophophate (NH_4_PF_6_), benzylbromide, 1-bromohexadecane, potassium iodide, Dopamine hydrochloride (Dop), Serotonin hydrochloride (Ser), Adrenaline hydrochloride (Adr) and Noradrenaline hydrochloride (Nor) were purchased from Sigma-Aldrich (Germany). Sodium sulfate (Na_2_SO_4_), 6N hydrochloric acid (HCl) and sulfuric acid (98%) were purchased from Scharlau (Spain). Sodium hydroxide pellets were purchased from Merck (Spain). Metallic sodium was purchased from Panreac (Spain). (6-{2-[2-(2-Methoxyethoxy)ethoxy]ethoxy}hexyl)trimethoxysilane (MTMS) was purchased from Sikemia (France). Sodium chloride (NaCl), sodium phosphate monobasic (NaH_2_PO_4_) and fetal bovine serum (FBS) were purchased from Thermo Fisher Scientific (UK). Potassium chloride (KCl), calcium chloride dihydrate (CaCl_2_∙2H_2_O), magnesium chloride hexahydrate (MgCl_2_∙6H2O), 4-(2-hydroxyethyl)piperazine-4-ethanesulfonic acid (HEPES), bovine serum albumin (BSA), sodium phosphate dibasic (Na_2_HPO_4_) and potassium phosphate monobasic (KH_2_PO_4_) were purchase from Sigma-Aldrich (UK). Trypsin 0.25% with ethylenediaminetetraacetic acid (EDTA) and McCOY’s 5A phenol red-free medium containing L-glutamine were purchased from Invitrogen (UK).

**S1.2. General methods**

Melting points were measured by CTP-MP 300 hot-plate apparatus with ASTM 2C thermometer using crystal capillaries purchased from Afora. ^1^H-NMR spectra were recorded on a Varian Gemini 300 spectrometer (300 MHz) and on a Varian Mercury 400 spectrometer (400 MHz) from *Centres Cientifics i Tecnològics de la Universitat de Barcelona* (CCiT-UB). ^13^C-NMR: Varian Mercury 400 (100 MHz) from CCiT-UB. NMR spectra were determined using CDCl_3_, (CD_3_)_2_SO or D_2_O as solvent with TMS (tetramethylsilane) as internal standard. Chemical shifts are expressed in parts per million (ppm) relative to the central peak of the solvent. Matrix Assisted Laser Desorption Ionization-Time of Flight Mass Spectrometry (MALDI-ToF-MS) analyses were performed using a Voyager-DE-RP mass spectrometer (Applied Biosystem, Framingham, USA) and High-resolution mass spectra (HRMS) were obtained by Electrospray (ESI) on a LC/MSD-ToF mass spectrometer (Agilent Technologies, 2006). MS analysis was operated in the delayed extraction mode using 2,5-dihydroxybenzoic acid (DHB) as a matrix. Thin layer chromatography (TLC) was performed on Merck silica gel plates coated with F254 fluorescence indicator. Column chromatography was carried out on silica gel 60 (Merck 9385, 230-400 mesh). Elemental analysis was performed on Thermo EA 1108 CHNS. UV-visible absorption spectra were obtained using an UV-1800 Shimadzu UV spectrophotometer, using quartz cuvettes with a 1 cm path length. Absorption spectra were determined in water or in a 1:1 DMSO/water solution. Infrared (IR) spectra were collected on a Thermo Nicolet Avatar 320 FT-IR spectrometer at room temperature in the range of 4000–400 cm^−1^, in KBr pellets (1% of the sample). Fluorescence excitation and emission spectra were recorded using a Hitachi F-4500 fluorescence spectrometer from UB. The spectra were recorded using quartz cuvettes with a 1 cm path length. Contact angles (ϴ) were measured in air with high purity deionized water by a 3 μL drop using a contact angle goniometer (THETALITE 100 with the software OneAttension, Finland), to determine the hydrophobicity of the modified surfaces. Values of the contact angle on at least three samples were measured to give statistical significance. Particle counting was performed approximately 100 μl of particles suspension into a Neubauer Chamber and counting the particles with an optical microscope. Fluorescence images were obtained with a Leica DMIRD microscope equipped with an inverted fluorescence microscope. The samples were observed in fluorescence and transmission light simultaneously. The UV-visible absorption spectroscopy was applied to determine the amount of incorporated porphyrin on **PSµCs**. The porphyrin conjugate was visualized by excitation of the fluorophore with a 552 nm laser diode, and the emitted fluorescence was detected between 600 and 700 nm. The presence of the different neurotransmitters was also confirmed using a fluorescence microscope equipped with a blue excitation filter (BP 450–490 nm) and an emission filter (LP 515 nm).

**S1.3. Synthesis and molecular characterization of 1, 2a, 2b, 5, 6, and 7**

***1,3-Bis(4-methyl-1-pyridiniomethyl)benzene dibromide*** ***(1).*** A solution of 4-methylpyridine (0.63 g, 6.76 mmol) in dry MeCN (13 mL) was added dropwise during 1 h to a solution of 1,3-bis(bromomethyl)benzene (0.89 g, 3.37 mmol) in dry MeCN (5 mL), and the mixture was heated under reflux for 1h. After cooling down to room temperature, the suspension was filtered off, and the white solid was washed with MeCN (5 mL) and dried to afford **1** (1.45 g, 97%): mp> 300 °C: ^1^H-NMR (300 MHz, (CD_3_)_2_SO, 25 °C): δ 9.09 (d, *J* = 6.6 Hz, 4H, H-2', 6'), 8.01 (d, *J* = 6.6 Hz, 4H, H-3', 5'), 7.61 (m, 4H, Ar-H-2, 4, 5, 6), 5.83 (s, 4H, N-CH_2_), 2.58 (s, 6H, CH_3_). ^13^C-NMR (100 MHz, (CD_3_)_2_SO, 25°C): δ 159.49 (Py-C 4'), 143.91 (Py-C2', 6), 135.47 (Ar-C 4, 6), 129.41 (Py- C 3', 5'), 128.28 (Ar- C 2, 4, 5, 6), 61.74 (N-CH_2_), 21.17 (CH_3_). MALDI-ToF-MS m/z: 605.6 (45 %) [M+DHB], 369.1 (30%) [M-Br]^+^, 350.1 (95%) [M-C_6_H_7_N]^+^, 289.1 (100%) [M-2Br]^+^, 276.0 (60 %) [M-(Br(C_6_H_7_N))]^+^. HMRS (ESI) m/z: (C_20_H_22_N_2_Br-2Br)^2+^ calculated 145.0884 found 145.0886. Elemental analysis (C_20_H_22_Br_2_N_2_): N (calculated 6.22 found 6.38) C (calculated 53.36 found 53.06) H (calculated 4.93 found 4.96) and IR spectrum (KBr, cm^-1^): 3017.44, 1632.01 and 1469.18.

***1,3-Bis(4-decyloxy-1-piridiniomethyl)benzene dibromide(2a).*** Metallic sodium (1.86 g, 80 mmol) was added in small portions to 1-decanol (12.66 g, 80 mmol) at 110 °C during 2 h. Then 4-chloropyridine hydrochloride (4.95 g, 33 mmol) was added and the mixture was stirred at 110 °C for 72 h. After cooling down to room temperature, water (50 mL) was added dropwise, and the solution was neutralized with HCl 6N solution. The aqueous phase was extracted with DCM (4 × 25 mL), and the organic phase was dried with anhydrous Na_2_SO_4_, filtered and the solvent was evaporated in vacuum. The residue was purified using a silica gel column chromatography using diethyl ether/ DCM (7:3) as eluent, followed by DCM/ MeOH (9:1), obtaining 4-decyloxypyridine (1.0 g, 13%).

A solution of 4-decyloxypyridine (450 mg, 1.91 mmol) in dry MeCN (30 mL) was added dropwise during 1h to a solution of 1,3-bis(bromomethyl)benzene (256 mg, 0.957 mmol) in dry MeCN (10 mL) and heated at 80 °C for 24 h. After cooling down to room temperature, the suspension was filtered off, and the white solid was washed with MeCN (5 mL) and dried to afford 2a (585 mg, 83%): mp >300 °C. ^1^H-NMR (300 MHz, CDCl_3_, 25 °C): δ 9.69 (d, *J* = 6.0 Hz, 4H, H-2', 6'), 8.32 (s, 1H, Ar-H2), 7.66 (d, *J* = 7.8 Hz, 2H, Ar-H4, 6), 7.35 (d, *J* = 6.0 Hz, 4H, H-3', 5'), 7.01 (dd, *J* = 7.5 Hz, 1H, Ar-H5), 5.93 (s, 4H, N-CH_2_), 4.22 (t, *J* = 5.45 Hz, 4H (O-CH_2_), 1.26 (s, 32H, 2(CH_2_)_8_), 0.87 (t, *J* = 5.4 Hz, 6H, CH_3_). ^13^C-NMR (100 MHz, CDCl_3_, 25°C): δ 170.35, (Py- C 4'), 146.83 (Py- C 2', 6'), 134.46 (Ar- C 1, 3), 131.34 (Ar- C2), 130.32 (Ar- C5), 130.12 (Ar-C 4, 6), 113.85 (Py- C 3', 5'), 71.31 (O-CH_2_), 60.92 (N-CH_2_), 31.75-22.62 ((CH_2_)_8_), 13.98 (CH_3_). MALDI-ToF–MS m/z: 727.4 (85%) [M-2Br+DHB]^+^, 655.3 (100%) [M-Br]^+^, 573.4 (20%) [M-2Br]^+^, 420.1 (5%) [M-(C_16_H_28_BrNO]^+^. HMRS (ESI) m/z: (C_38_H_58_N_2_O_2_Br_2_-2Br)^2+^ calculated 287.2235 found 287.2244, (C_38_H_58_N_2_O_2_Br_2_-Br)^+^ calculated 653.3667 found 653.3676. Elemental analysis (C_38_H_58_Br_2_N_2_O_2_·1H_2_O): N (calculated 3.72 found 3.69) C (calculated 60.64 found 60.60) H (calculated 8.03 found 8.00) and IR spectrum (KBr, cm^-1^): 2917.03, 1642.25 and 1467.80.

***1,3-Bis(4-octadecyloxy-1-pyridiniomethyl)benzene dibromide (2b).*** Metallic sodium (1.86 g, 80 mmol) was added to 1-octadecanol (21.9 g, 80 mmol) at 110 °C for 2 h. Then 4-chloropyridine hydrochloride (4.95 g, 33 mmol) was added and the mixture was stirred for 72 h at 110 °C. After cooling down to room temperature, water (50 mL) was added dropwise, and the solution neutralized with HCl 6N. The aqueous phase was extracted with DCM (4 × 25 mL), and the organic phase was dried with anhydrous Na_2_SO_4_ and the solvent were evaporated in vacuum. The residue was purified using a silica gel column chromatography using diethyl ether/ DCM (7:3) as eluent, following by column chromatography in DCM/ MeOH (9:1), obtaining 4-octadecyloxy-pyridine (2.66 g, yield 23%).

A solution of 4-octadecyloxy-pyridine (150 mg, 0.43 mmol) in dry MeCN (13 mL) was added dropwise during 1 h to a solution of 1,3-bis(bromomethyl)benzene (57 mg, 0.22 mmol) in dry MeCN (10 mL) and heated at 75 °C for 24 h. After cooling down to room temperature, the suspension was filtered off, and the white solid was washed with MeCN (5 mL) and dried to afford **2b** (1.80 g, 94%): mp> 300 °C. ^1^H-NMR (300 MHz, CDCl_3_, 25 °C): δ 9.76 (d, *J* = 7.5 Hz, 4H, H- 2', '6), 8.39 (s, 1H, Ar-H2), 7.69 (d, *J* = 6.3 Hz, 2H, Ar-H 4, 6), 7.33 (d, *J* = 7.5 Hz, 4H, H- 3', 5'), 7.01 (dd, *J* = 7.8 Hz, 1H, Ar-H5), 5.92 (s, 4H, N-CH_2_), 4.21 (t, *J* = 6.45 Hz, 4H, (O-(CH_2_)), 1.25 (s, 64H, 2(CH_2_)16), 0.87 (t, *J* = 6.6 Hz, 6H, (CH_3_)). ^13^C-NMR (100 MHz, CDCl_3_, 25 °C): δ 170.35, (Py- C 4'), 146.95 (Py- C 2', 6'), 134.49 (Ar- C 1, 3), 131.26 (Ar- C2), 130.32 (Ar- C5), 130.07 (Ar- C 4, 6), 113.89 (Py- C 3', 5'), 71.40 (O-CH_2_), 61.25 (N-CH_2_), 31.83-22.6 ((CH_2_)_16_), 14.09 (CH_3_). MALDI-ToF–MS m/z: 879.7 (100%) [M-Br]^+^, 797.7 (85%) [M-2Br]^+^, 530.37 (30%) [M-Br(C_23_H_41_NO)]^+^, 450.47 (20%) [M-((2Br)C_23_H_41_NO)]^+^. HMRS (ESI) m/z: (C_54_H_90_N_2_O_2_B_r2_ -2Br)^2+^ calculated 399.3489 found 399.3496. Elemental analysis (C_54_H_90_Br_2_N_2_O_2_·1H_2_O): N (calculated 2.92 found 3.01) C (calculated 66.38 found 66.81) H (calculated 9.49 found 9.58) and IR spectrum (KBr, cm^-1^): 2917.62, 1642.80 and 1467.65.

***1,3-Bis(4-methyl-1-pyridiniomethyl)-5-propargyloxybenzene dibromide (5).*** The three precursors of 5 (dimethyl-5-propargyloxyisophtalate, 1,3-dihydroxymethyl-5-propargyloxybenzene and 1,3-dibromomethyl-5-propargyloxybenzene) were synthetized following protocols previously reported. ^1^

A solution of 4-methylpyridine (0.63 g, 6.7 mmol) in dry MeCN (13 mL) was stirred for 1 h at 75 °C under argon atmosphere. After this time a solution of 1.3-dibromomethyl-5-propargyloxybenzene (1.16 g, 1.89 mM) in dry MeCN (5 mL) was added to the flask and stirred overnight. After cooling down to room temperature, the suspension was filtered off, and the white solid was washed with MeCN (5 mL) and dried to afford **5** (1.62 g, 97%): mp=270 °C: ^1^H-NMR (400 MHz, D_2_O, 25 °C): δ 8.54 (d, *J* = 6.4 Hz, 4H), 7.76 (d, *J* = 5.9 Hz, 4H), 6.97 (s, 3H), 5.59 (s, 4H), 6.67 (s, 2H), 2.54 (s, 7H). ^13^C-NMR (100 MHz, D_2_O, 25 °C): δ 160.9 (Ar-C 1), 158.1 (Py-C 3), 143.1 (Py-C 1,5), 135.9 (Ar-C 3, 5), 128.8 (Py-C 2, 4), 122.0 (Ar-C 4), 116.3 (Ar- C 2, 6), 78.0 (O-CH_2_CCH), 77.0 (O-CH_2_CCH), 62.6 (N-CH_2_), 56.1 (O-CH_2_CCH), 21.1 (CH_3_). MALDI-ToF-MS m/z: 488.1 (28%) [M-CH_3_]^+^, 423.1 (20%) [M-Br]^+^, 343.1 (100%) [M-2Br]^+^, 329.9 (90%) [M-2Br (CH_3_)]^+^. HMRS (ESI) m/z: (C_23_H_24_N_2_O-2Br)^+^ calculated 344.1878 found 343.1813. IR spectrum (KBr, cm^-1^): 2103, 1638 and 1466.

***1,3-Bis(4-octadecyl-1-pyridiniomethyl)-5-propargyloxybenzene dibromide (6).*** Metallic sodium (0.19 g, 8 mmol) was added to 1-octadecanol (2.19 g, 8 mmol) at 110 °C for 2 h. Then 4-chloropyridine hydrochloride (0.49 g, 3.3mmol) was added and the mixture was stirred for 72 h at 110 °C. After cooling down to room temperature, water (15 mL) was added dropwise, and the solution was neutralized with 6N HCl solution. The aqueous phase was extracted with DCM (4 × 15 mL), and the organic phase was dried with anhydrous Na_2_SO_4_, filtered and the solvent was evaporated in vacuum. The residue was purified using a silica gel column chromatography using diethyl ether/ DCM (7:3) as eluent, following by DCM/MeOH (9:1), obtaining 4-octadecyloxypyridine (0.27 g, yield 25%).

A solution of 4-octadecyloxy-pyridine (50 mg, 0.14 mmol) in dry MeCN (5 mL) was stirred for 1 h at 55 °C under argon atmosphere. After this time a solution of 1.3-dibromomethyl-5-propargyloxybenzene (22.1 mg, 0.07 mmol) in dry MeCN (5 mL) was added to the flask and stirred for 48 h at 80 °C. After cooling down to room temperature and the solvent were evaporated in vacuum. The white solid was washed with MeCN (10 mL) and dried to afford compound **6** (67 mg, 96%): mp= 200 °C. ^1^H-NMR (400 MHz, CDCl_3_, 25 °C): δ 9.68 (d, *J* = 8.0 Hz, 4H, H 2', '6), 7.88 (s, 1H, Ar-H2), 7.32 (d, *J* = 7.3 Hz, 2H, Ar-H 4, 6), 7.30 (d, *J* = 8.0 Hz, 4H, H 3', 5'), 7.21 (s, 1H, Ar-H5), 5.82 (s, 4H, N-CH_2_), 4.31 (d, *J* = 7.0 Hz, 2H, -OCH_2_CCH), 4.16 (t, *J* = 6.0 Hz, 4H, O-CH_2_), 2.50 (t, *J* = 6.0 Hz, 1H, -CCH) 1.79-1.72 (m, 4H, 2CH_2_), 1.33-1.26 (m, 4H, 2CH_2_), 1.16 (s, 56H, 2(CH_2_)_14_), 0.81 (t, *J* = 6.5 Hz, 6H, 2CH_3_). ^13^C-NMR (100 MHz, CDCl_3_, 25°C): δ 170.5, (Py- C (4')), 158.1 (Ar- C 6), 147.0 (Py- C 2', 6'), 135.9 (Ar- C2, 4), 124.1 (Ar- C3), 116.9 (Ar-C 1,4), 114.0 (Py- C 3',5'), 77.8 (O-CH_2_CCH), 76.3 (O-CH_2_CCH), 71.4 (O-CH_2_), 61.1 (N-CH_2_), 61.1 (O-CH_2_CCH), 31.89 (CH_2_), 29.6-29.2 (CH_2_)14, 28.3 (CH_2_), 25.6 (CH_2_), 22.6 (CH_2_), 14.1 (CH_3_). MALDI-ToF–MS m/z: 930.7 (100%) [M-Br]^+^. MRS (ESI) m/z: (C_57_H_92_N_2_O_3_Br_2_ -2Br-H)^2+^ calculated 425.3710 found 426.3556. IR spectrum (KBr, cm^-1^): 2917, 1642 and 1466.

***1,3-Bis(1-methylimidazoliomethyl)-5-propargyloxybenzene dibromide (7).*** A solution of 1,3-dibromomethyl-5-propargyloxybenzene (1.38 g, 4.3 mmol) in dry MeCN (5 ml) was added to a solution of 1-methylimidazole (0.71 g, 8.6 mmol) in dry MeCN (13 ml) and stirred for 24 h at 80 °C. After cooling down to room temperature, the suspension was filtered off, and the white solid was washed with MeCN (5 mL) and dried to afford compound **7** (1.73 g, 84%): mp=110 °C, ^1^H-NMR (400 MHz, D_2_O, 25 °C): δ ppm 3.73 (t, *J* = 1.0 Hz, 1H), 3.91 (s, 6H), 4.83 (s, 2H), 5.40 (s, 4H), 7.08-7.04 (m, 3H), 7.46 (dd, *J* = 8.1 Hz, *J* = 8.0 Hz, 6H). ^13^C NMR (100 MHz, D_2_O, 25 °C): δ 35.9 (CH_3_), 52.1 (N-CH_2_), 56.0 (O-CH_2_CCH), 77.0 (O-CH_2_CCH), 78.1 (O-CH_2_CCH), 115.7 (Ar-C 2, 6), 121.4 (Ar-C 4) 122.0 (Im-C 3), 123.8 (Im-C2), 136.1 (Im-C1), 158.0 (Ar-C1). MALDI-ToF-MS m/z: 401.0 (90%) [M- Br]^+^, 321.1 (100 %) [M-2Br]^+^, 241.1 (60%) [M-2Br (C_4_H_6_N_2_)]^+^. HMRS (ESI) m/z: (C_19_H_22_N_4_O-2Br)^+^ calculated 322.1800 found 321.1707. IR spectrum (KBr, cm^-1^): 3281, 2360 and 1467.

**S1.4. Surface activation of PWs, PWµCs, or PSµCs**

The **PWs** (or **PWµCs**) with an area of 1 cm^2^ were immersed in a freshly prepared mixture of H_2_SO_4_ (98%) and H_2_O_2_ (30%) known as Piranha solution, at a volume ratio of 1.4 mL and 0.6 mL, respectively (in a 7:3 ratio) for 1 hour at room temperature. Subsequently, the **PWs** were rinsed with 3 mL of water five times. Freshly prepared **PWs** were then submerged in an alkaline mixture consisting of NH_4_OH (20%), H_2_O_2_ (30%), and distilled H_2_O, at a volume ratio of 0.3 mL, 0.3 mL, and 1.4 mL, respectively (in a 1:1:5 ratio) for 30 minutes to activate the wafers. Afterward, the **PWs** were thoroughly rinsed with 3 mL of water five times and dried with nitrogen.

For surface activation of **PSµCs**, a mixture of H_2_SO_4_ (98%) and H_2_O_2_ (30%) (Piranha solution), at a volume ratio of (0.7 mL, 0.3 mL, respectively) (7:3) was added to a microtube, containing approximately 10^6^ **PSµCs**, and the **PSµCs** were incubated for 1 h at room temperature and stirred on a continuous shaking at 400 rpm. Afterwards, the suspensions were centrifuged for 15 minutes at 13500 rpm and washed (2 × 1 mL) with water. Freshly prepared alkaline mixture of NH_4_OH (20%), H_2_O_2_ (30%), and distilled H2O were added to wafers in a volume ratio of 0.1 mL, 0.1 mL, and 0.7 mL (1:1:5), respectively, for 30 min to activate the **PSµCs** followed by centrifugation of suspensions for 15 min at 13500 rpm and washing (2 × 1 mL) with water.

**S1.5. Preparation of control samples for PWs, PWµCs, or PSµCs**

For the non-covalent functionalization of **PWs** or **PWµCs** with pyridinium **1** and **2**, imidazolium salts **3** and **4**, and bis-bipyridinium salts **9-11**, the **PWs** used as controls were solely immersed in a solution (2 mL) of each respective compound in its corresponding solvent (**1** or **3** in water, **2a**, **2b**, or **4** in chloroform, **9**, **10**, or **11** in DMSO). Furthermore, a control of **PWs** (or **PWµCs**) was examined, in which the substrate was solely immersed in the solvent used at each step of this functionalization process.

For covalent functionalization of **PWs** (or **PWµCs**) with pyridinium **5** and **6** and imidazolium salts **7** and **8**, the **PWs** used as controls were solely immersed in a solution (2 mL) of each respective compound in its corresponding solvent (**5** or **6** in DMSO, **7** in water, and **8** in DMSO).

For the non-covalent functionalization of **PSµCs** with pyridinium **2b**, imidazolium salts **4**, and bis-bipyridinium salt **9**, the microtubes containing **PSµCs** used as control in the non-covalent immobilization of pyridinium **2** and imidazolium salts **4** were only immersed in a solution of each of these compounds in CHCl_3_ (2 mL). Additionally, a control of **PSµCs** was analysed, where the substrate was only immersed in the solvent used in each step of functionalization.

For the covalent functionalization of **PSµCs** with pyridinium **6** and imidazolium salts **8**, the microtubes containing **PSµCs** were also immersed in the solvent utilized in each step of this functionalization, as well as only solutions of pyridinium **6** and imidazolium salts **8** in DMSO (1 mL) as a control.

For the immobilization of the porphyrin **Na-ZnTCPP** into functionalized **PWs** and **PWµCs**, and **PSµCs**, the **PWs** (or **PWµCs**) used as controls in the incorporation of **Na-ZnTCPPP** as a photosensitizer were solely immersed in an aqueous solution (2 mL) of this porphyrin in water. In the case of **PSµCs**, the **PSµCs** used as control in the incorporation of **Na-ZnTCPP** were only immersed in an aqueous solution (1 mL) of this porphyrin.

For the immobilization of neurotransmitters Dop, Ser, Adr, and Nor on **PWs** or **PWµCs**, The **PWs** (or **PWµCs**) used as control in the immobilization of bis-bipyridinium salts **9**, **10** or **11** and the subsequent incorporation of the different neurotransmitters Dop, Ser, Adr or Nor were separately immersed in a solution of each of these compounds in the corresponding solvent (DMSO (2 mL) for the bis- bipyridinium salts and H_2_O (2 mL) for the neurotransmitters). Additionally, a control of wafers was analyzed, where the substrate was only immersed in the solvent used in each step of this functionalization. In the case of **PSµCs**, non-functionalized **PSµCs** were also incubated in solution of the neurotransmitters Dop or Ser which H_2_O was the solvent.


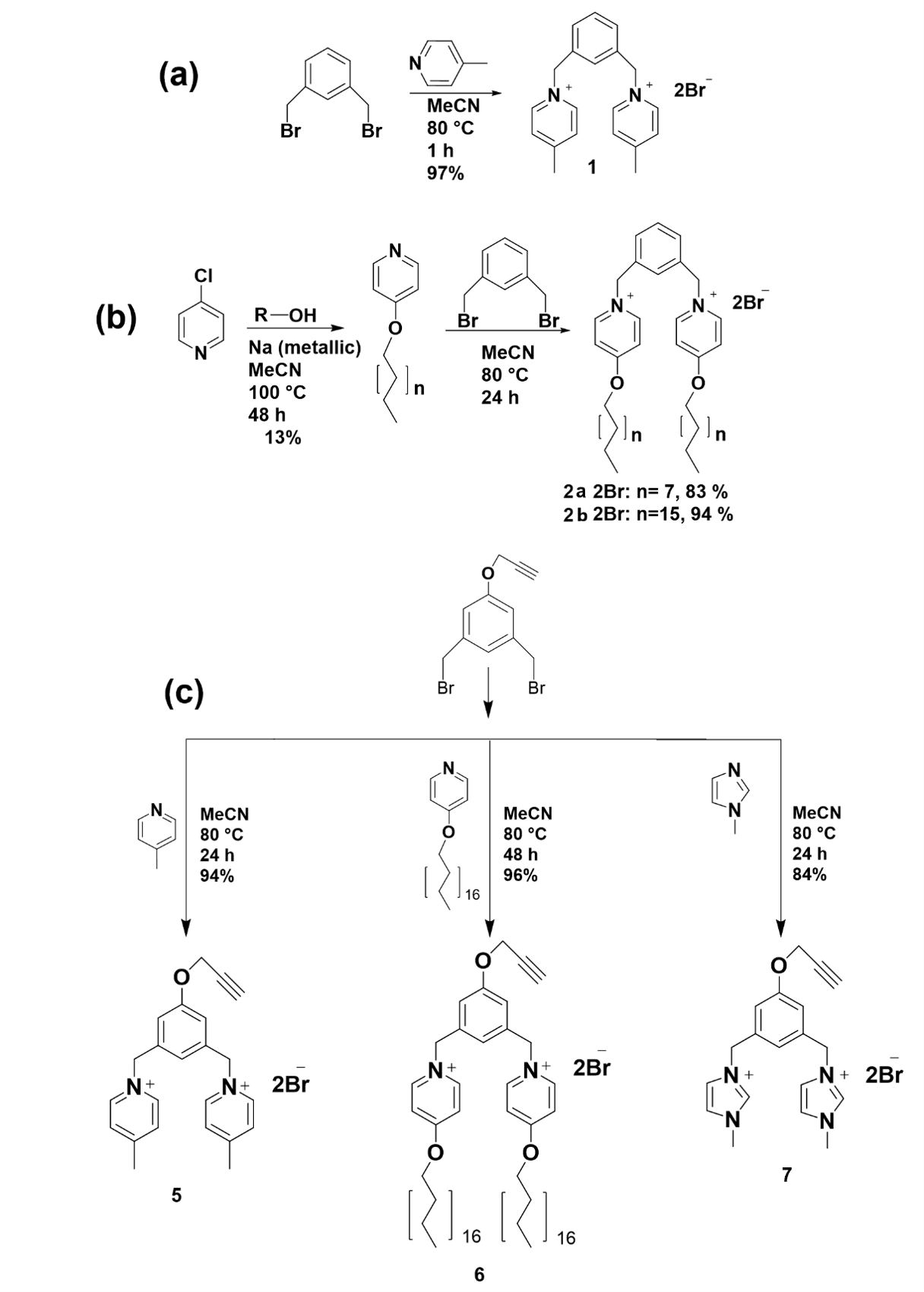


Scheme S1. Synthesis of the pyridinium salts a) 1, b) 2a and 2b, c) pyridinium salts 5 and 6 and imidazolium salts 7 used in the functionalization of the polysilicon substrates ((PWs, PWµCs, and PSµCs).


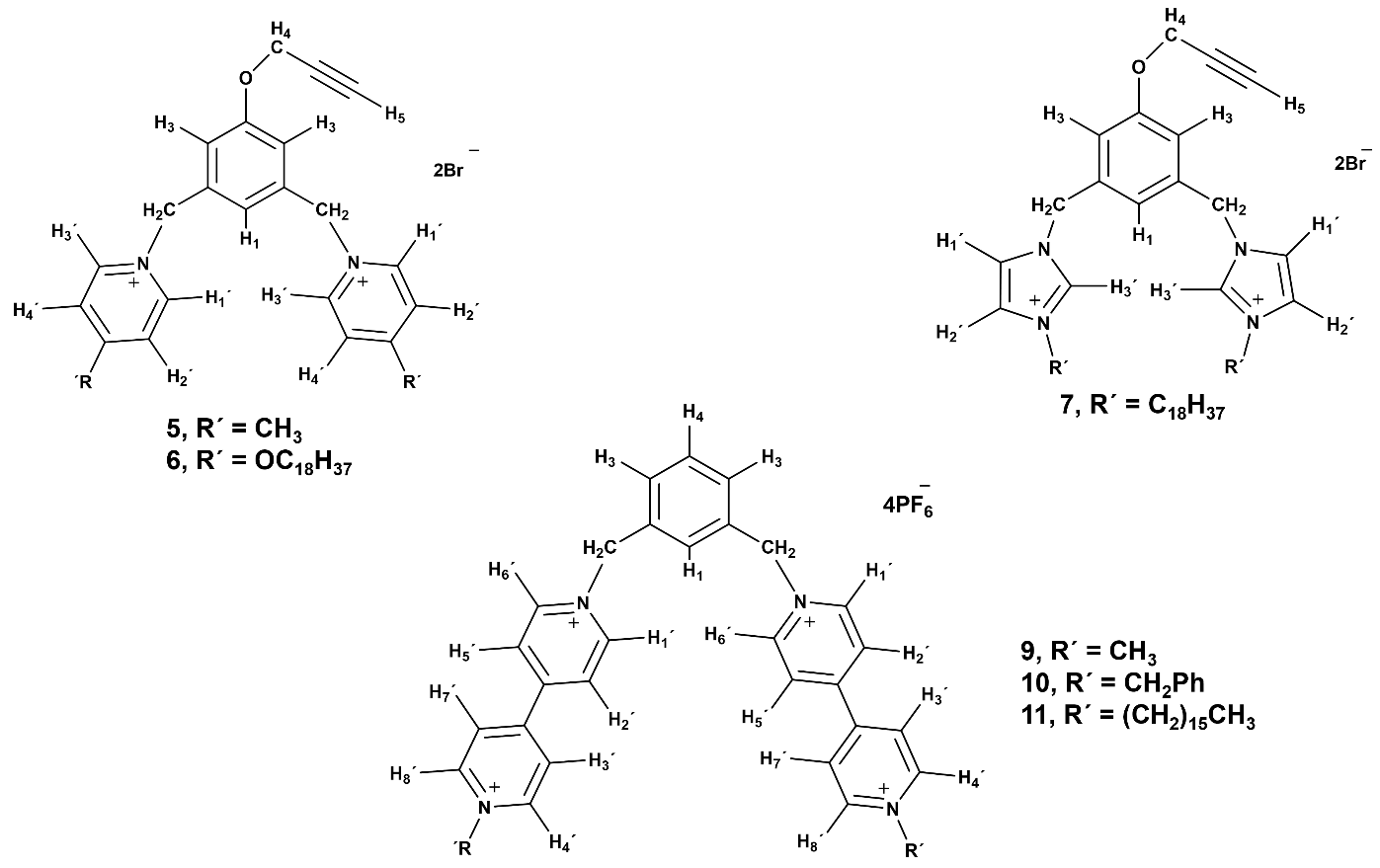


**Figure S1**. Numbering of the hydrogen atoms in the structures of the compounds **5-7 and 9-11**.

**Figure S2.** ^1^H-NMR spectrum of **1** recorded in (CD_3_)_2_SO at 300 MHz.

**Figure S3.** ^13^C-NMR spectrum of **1** recorded in (CD_3_)_2_SO at 100 MHz.


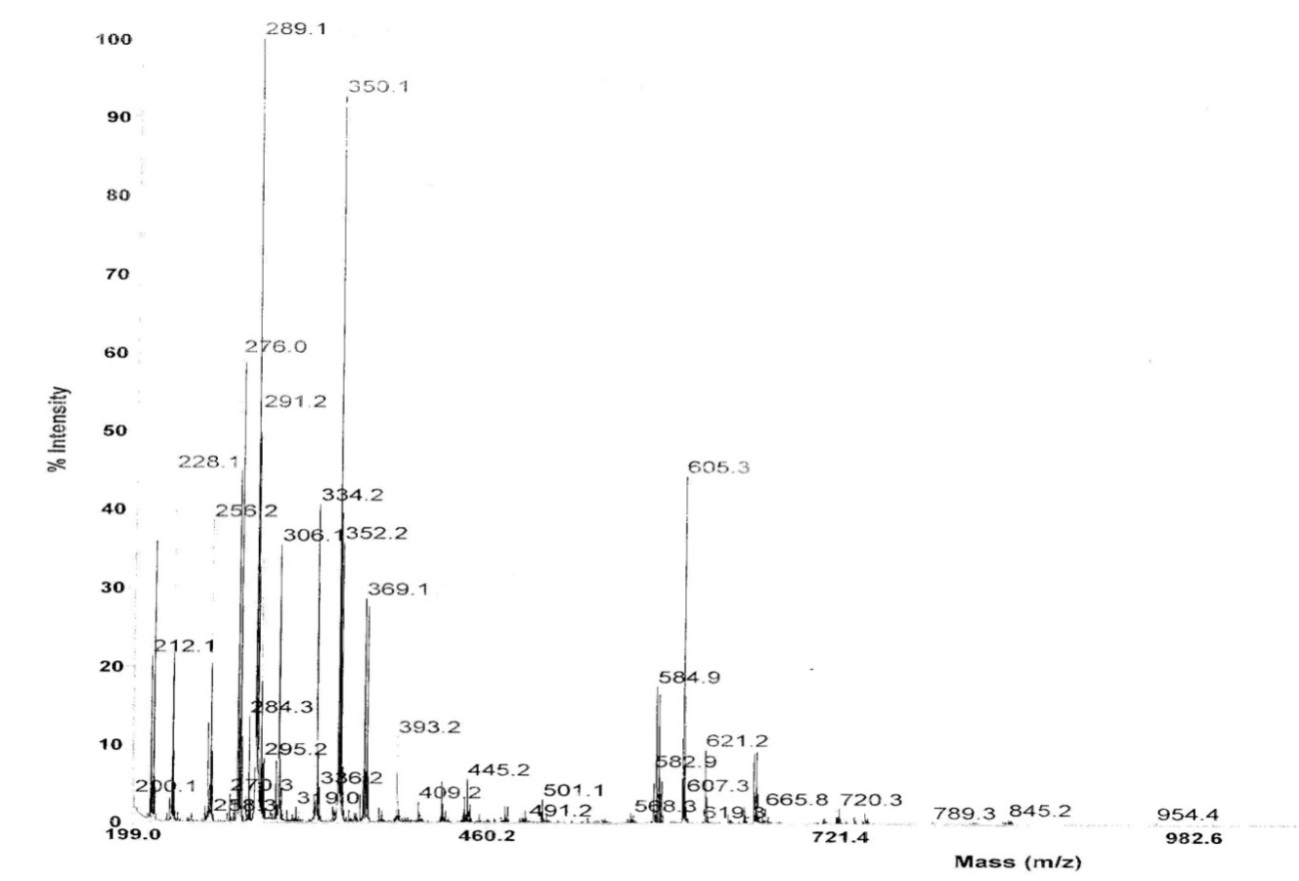


**Figure S4.** MALDI-ToF-MS (m/z) spectrum of **1** with matrix DHB.


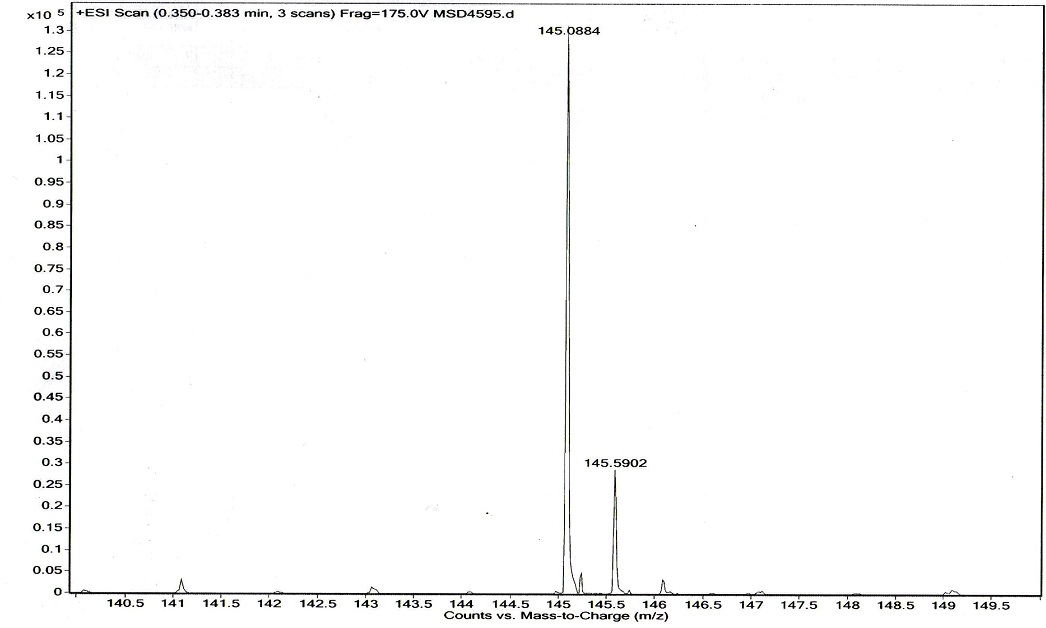


**Figure S5.** HMRS-ESI spectrum (m/z) spectrum of **1**.


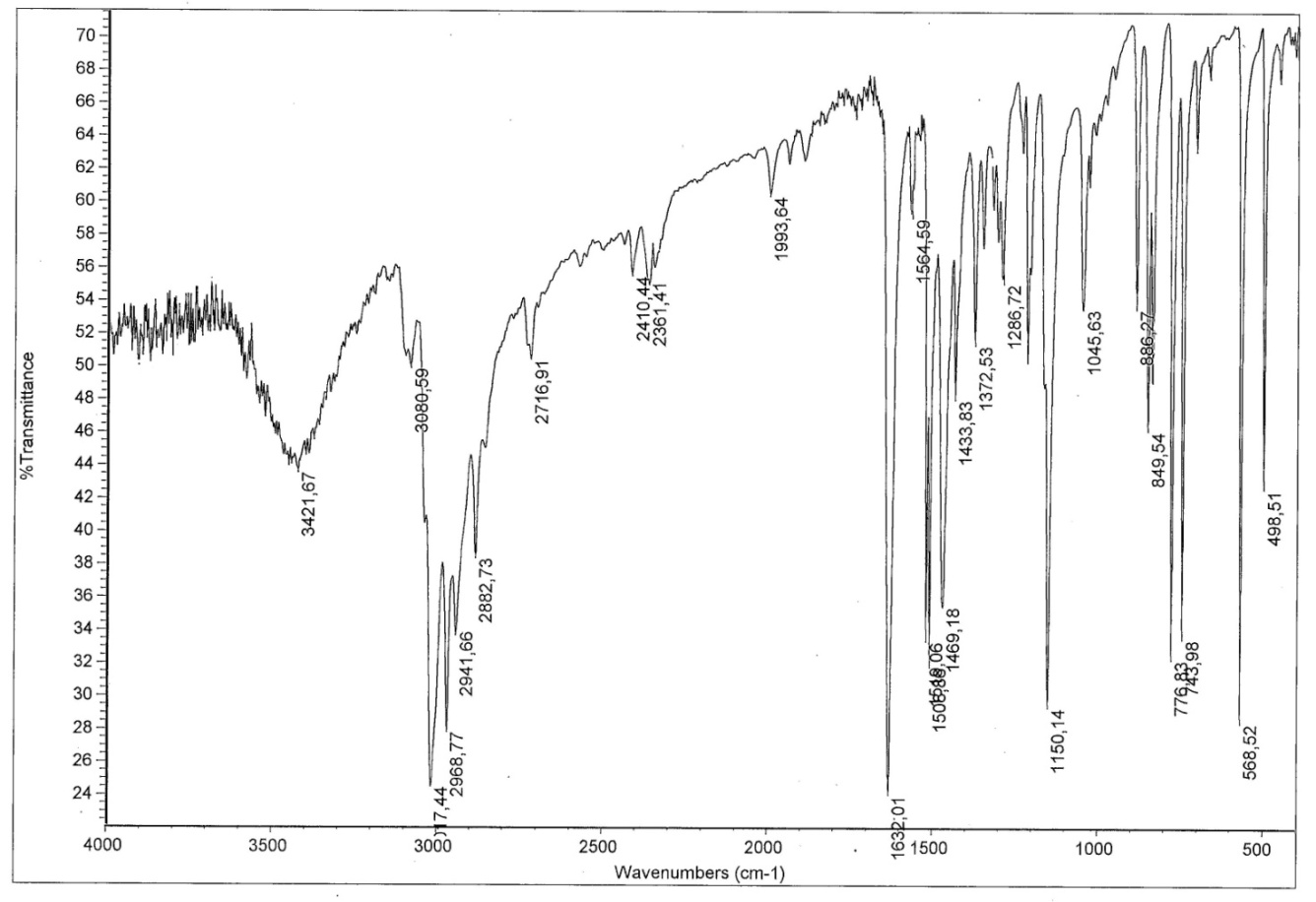


**Figure S6.** IR spectrum of **1** in KBr.

**Figure S7.** ^1^H-NMR spectrum of **2a** recorded in CDCl_3_ at 300 MHz.

**Figure S8.** ^13^C-NMR spectrum of **2a** recorded in CDCl_3_ at 100 MHz.


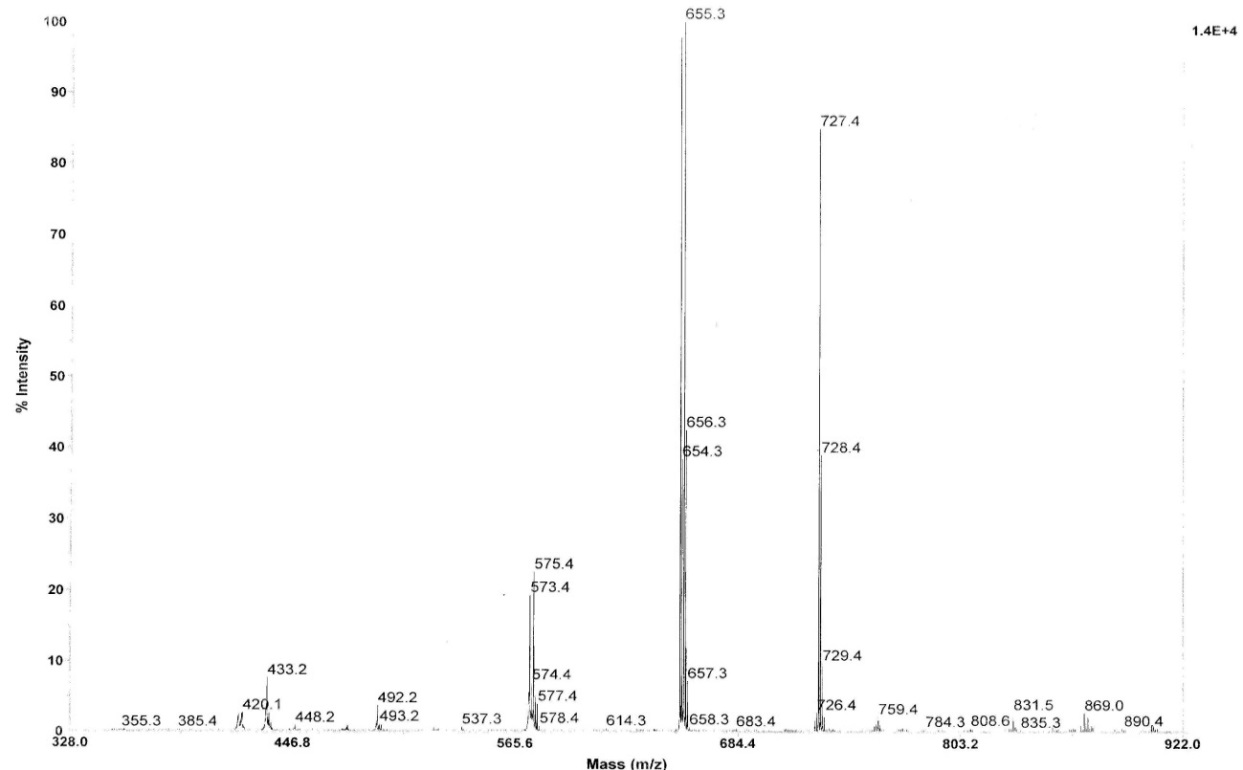


**Figure S9.** MALDI-ToF-MS (m/z) spectrum of **2a** with matrix DHB.

**
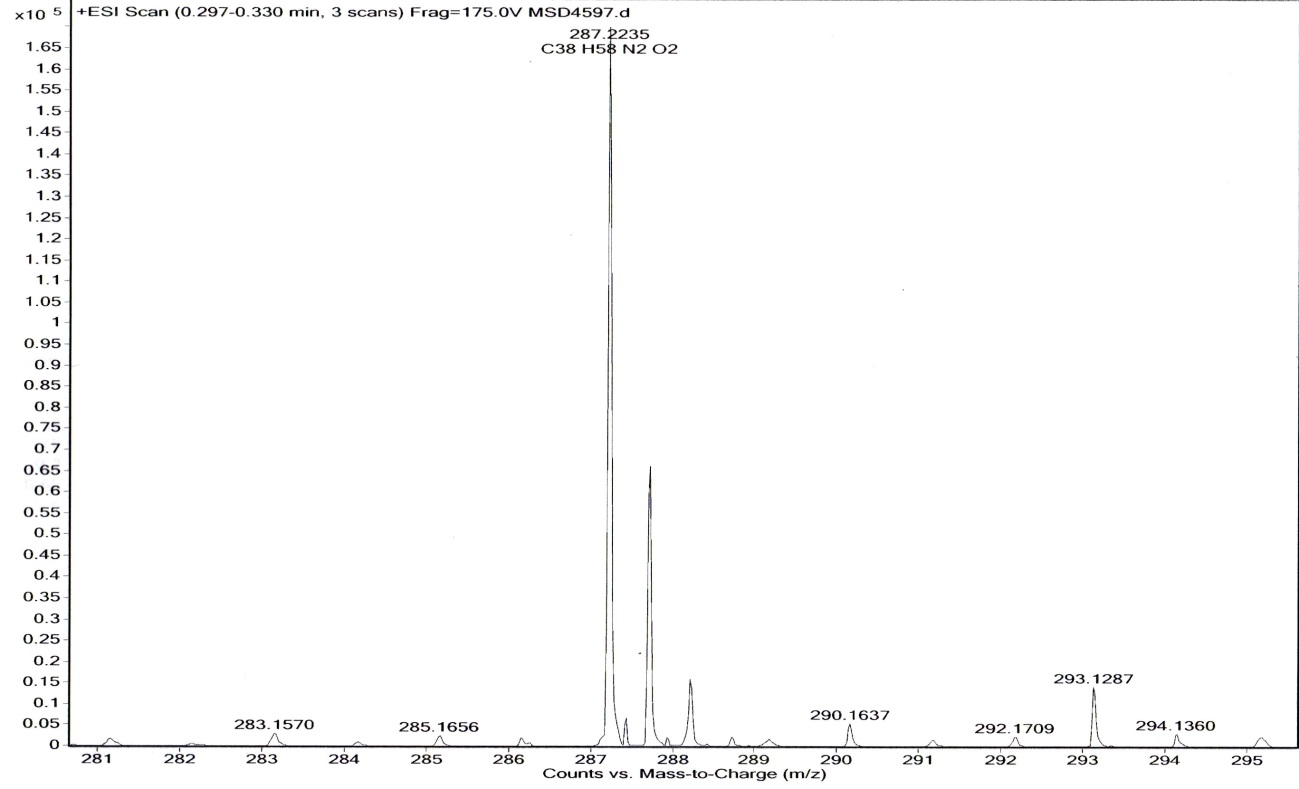
**

**Figure S10.** HMRS-ESI spectrum (m/z) spectrum of **2a**.


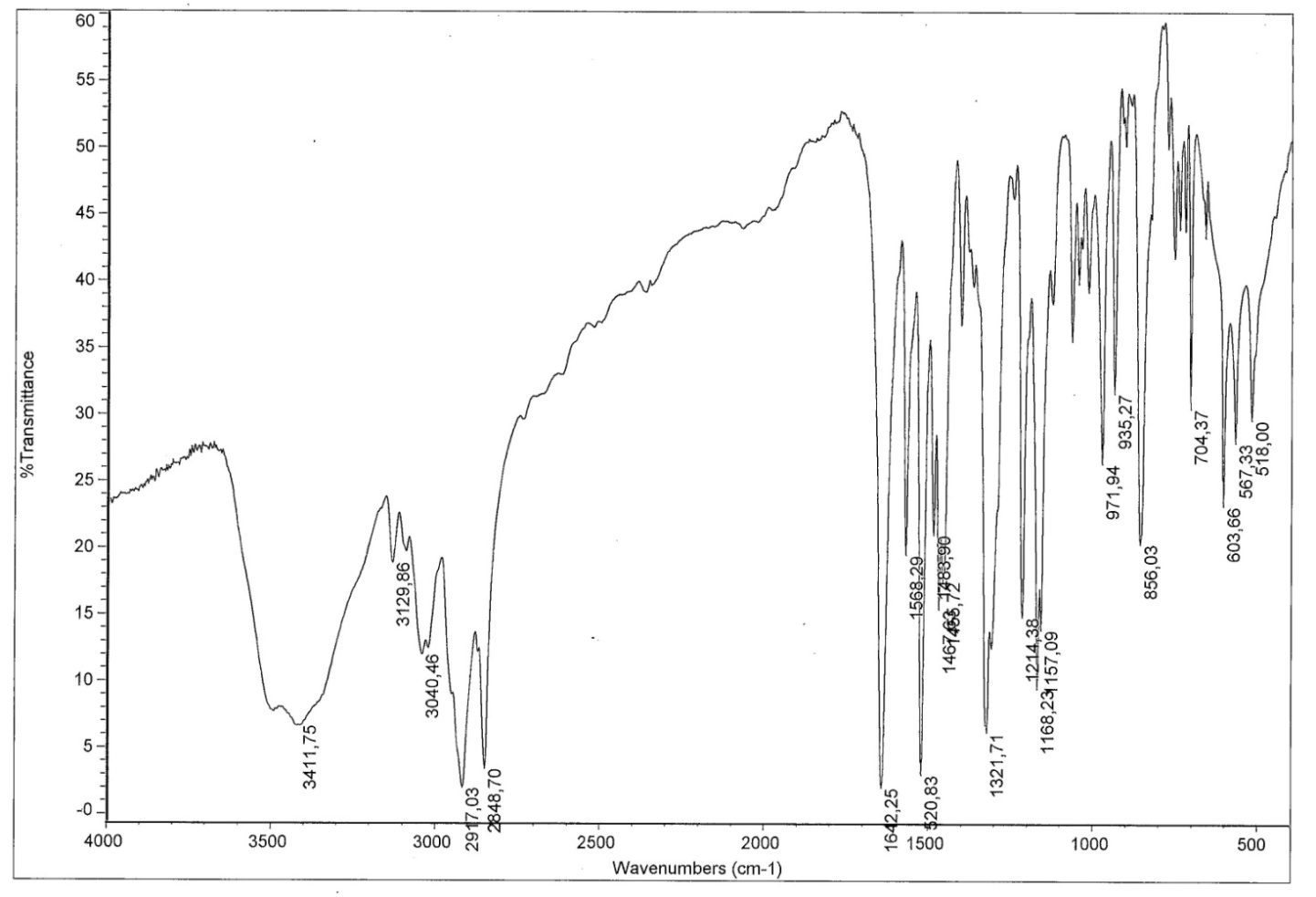


**Figure S11.** IR spectrum of **2a** in KBr.

**Figure S12.** ^1^H-NMR spectrum of **2b** recorded in CDCl_3_ at 300 MHz.

**Figure S13.** ^13^C-NMR spectrum of **2b** recorded in CDCl_3_ at 100 MHz.


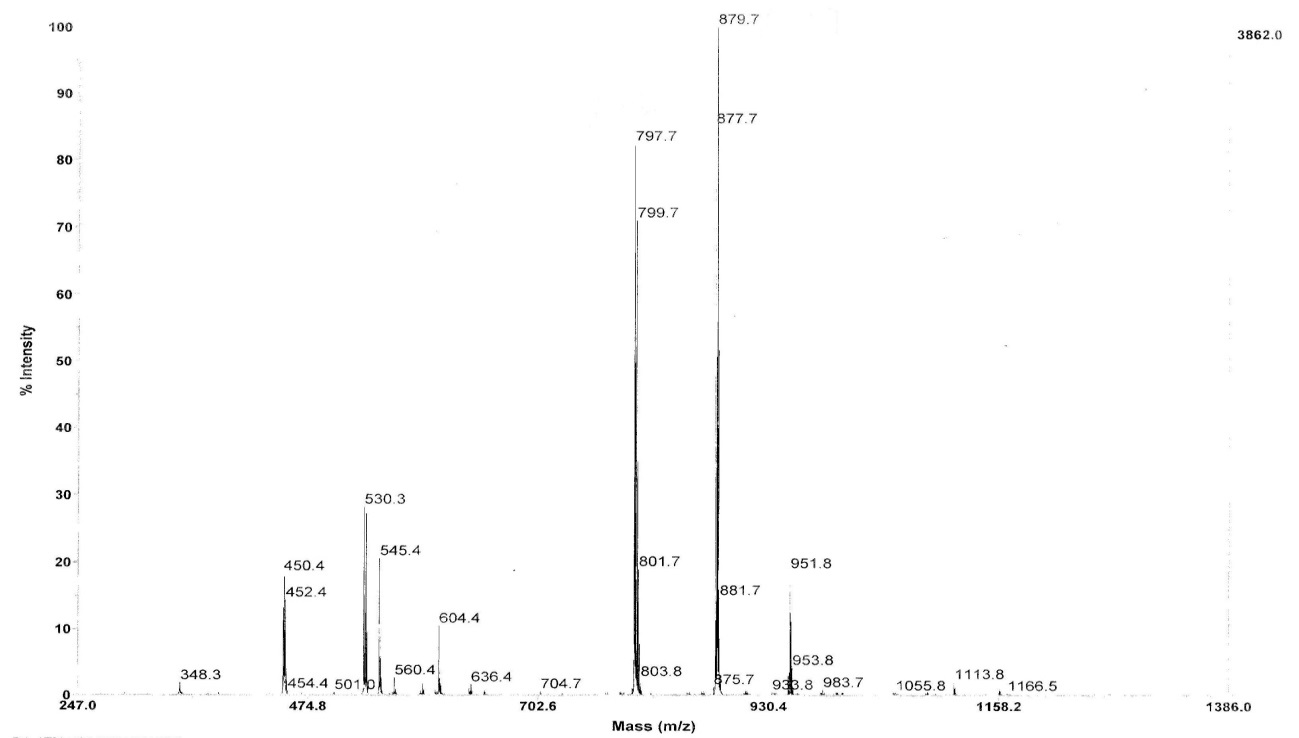


**Figure S14.** MALDI-ToF-MS (m/z) spectrum of **2b** with matrix DHB.


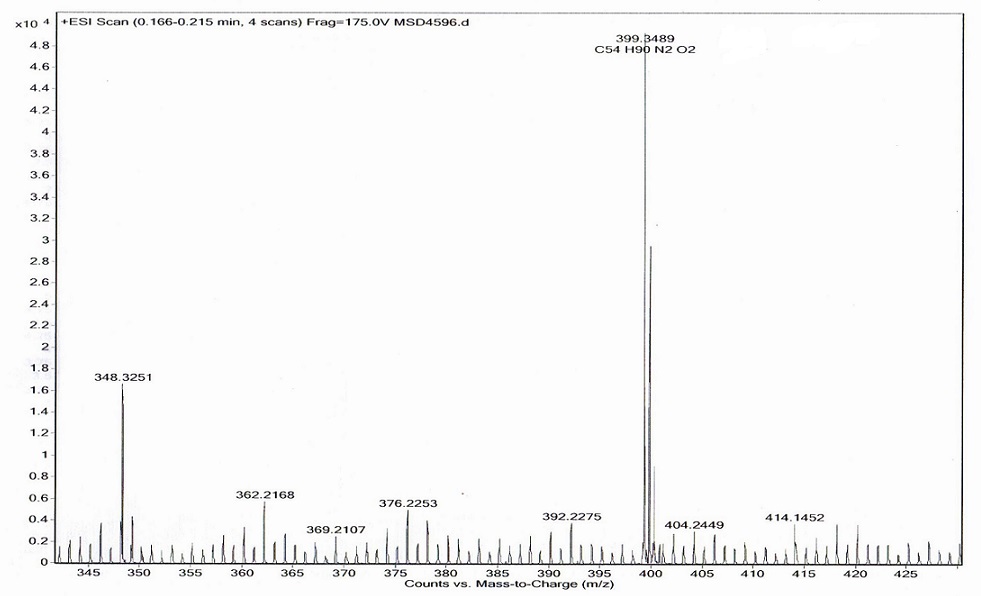


**Figure S15.** HMRS-ESI spectrum (m/z) spectrum of **2b.**


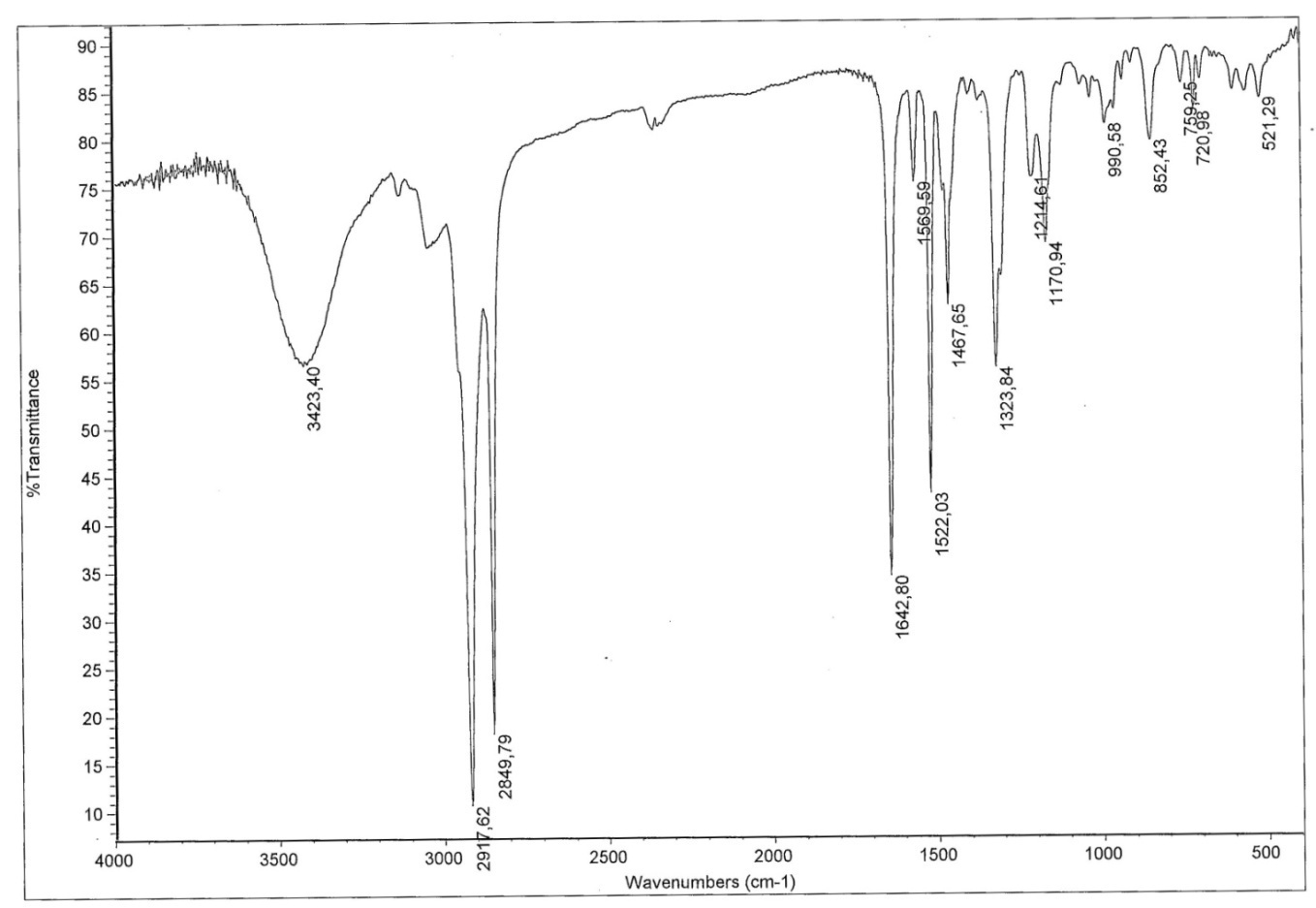


**Figure S16.** IR spectrum of **2b** in KBr

**
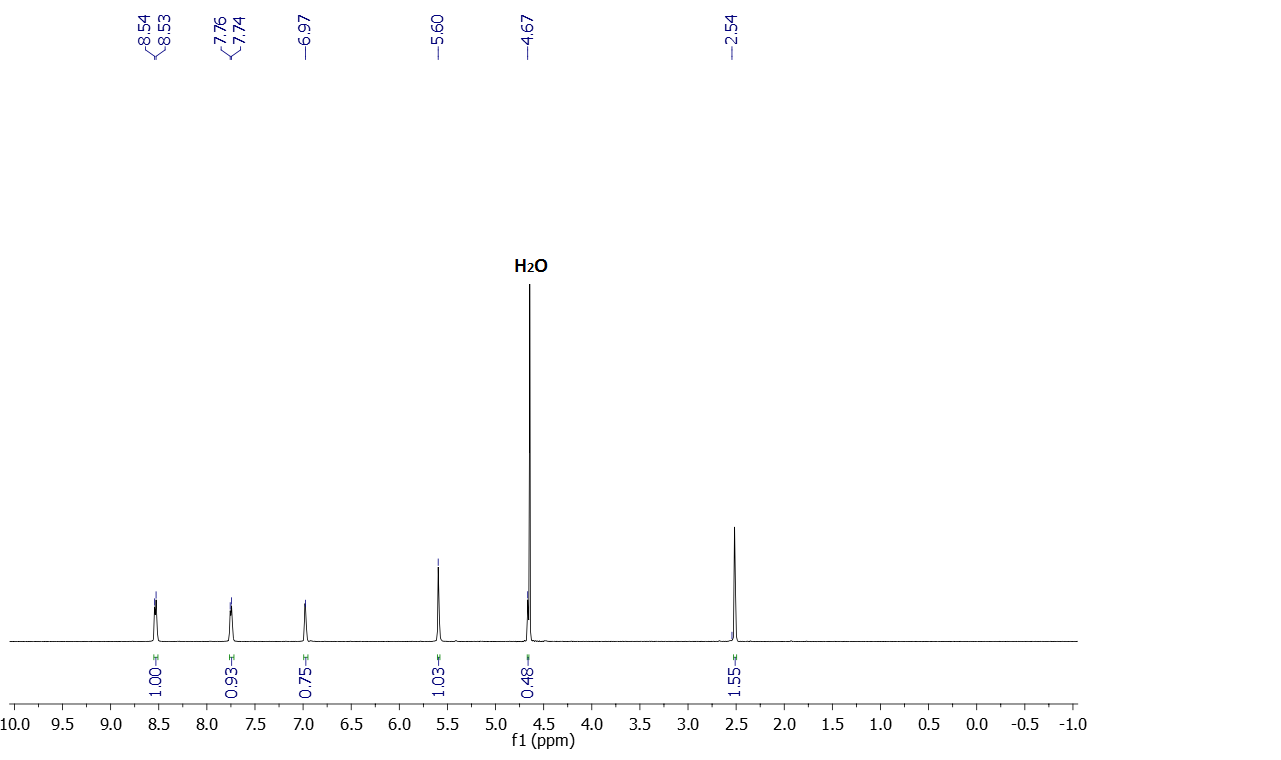
**

**Figure S17.** ^1^H-NMR spectrum of **5** recorded in D_2_O at 400 MHz

**Figure S18**. ^13^C-NMR spectrum of **5** recorded in D_2_O at 100 MHz.

**
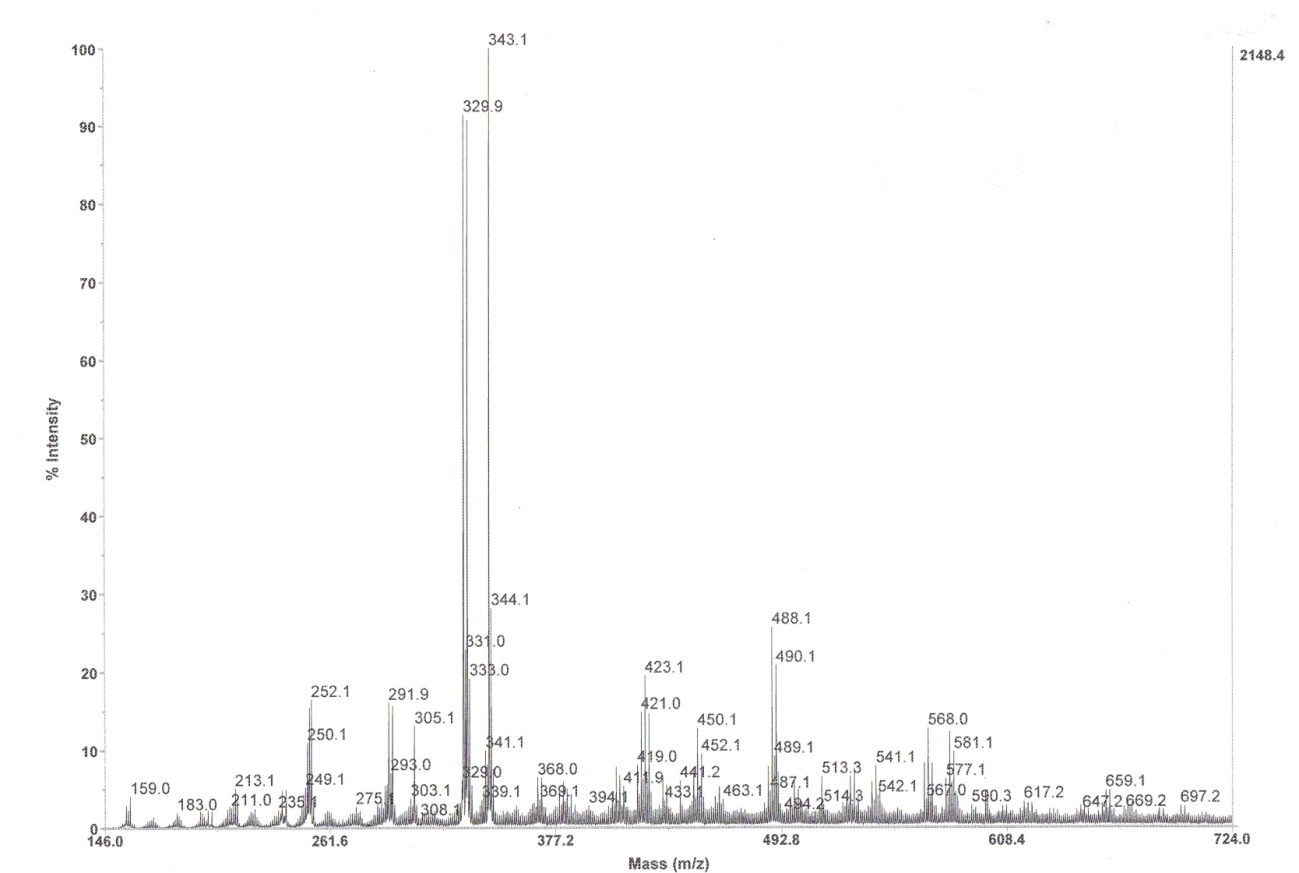
**

**Figure S19**. MALDI-ToF-MS (m/z) spectrum of **5** with DHB as matrix

**
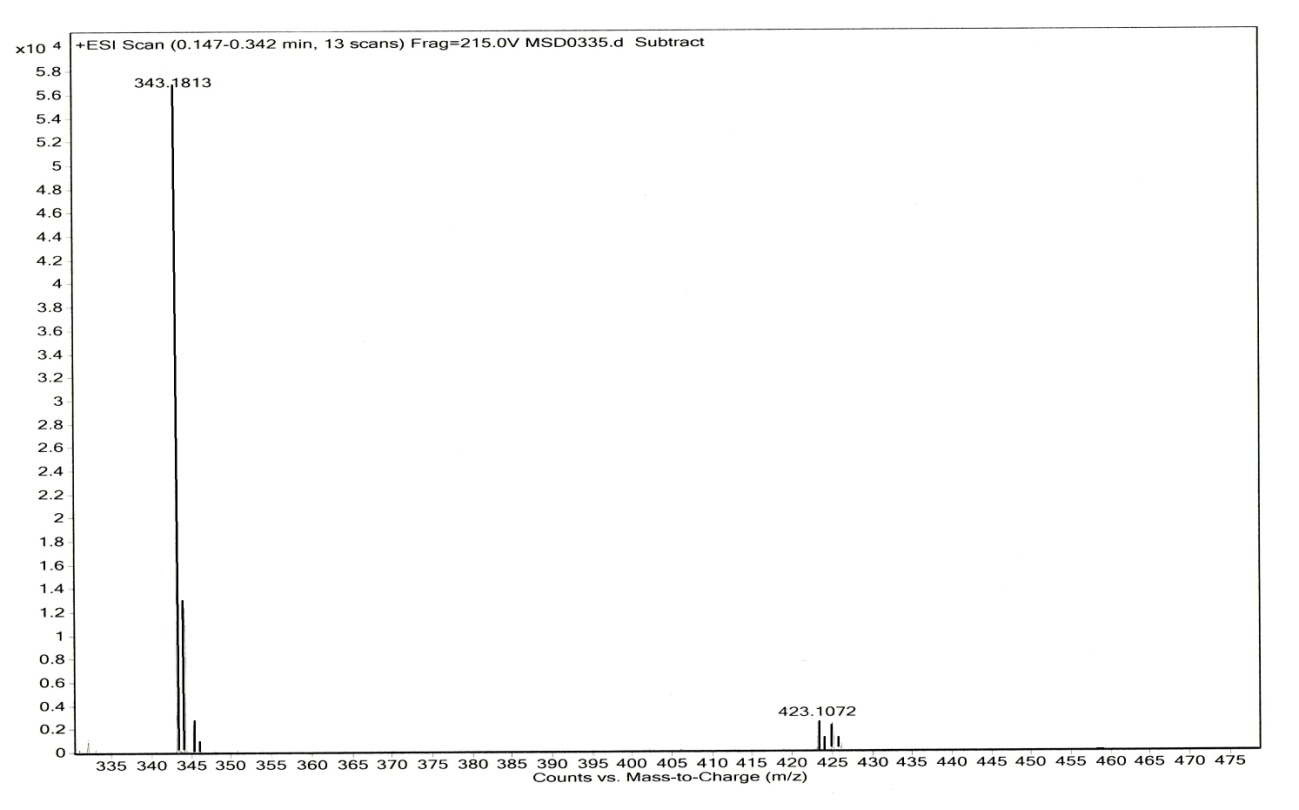
**

**Figure S20**. HMRS-ESI spectrum (m/z) spectrum of **5.**

**
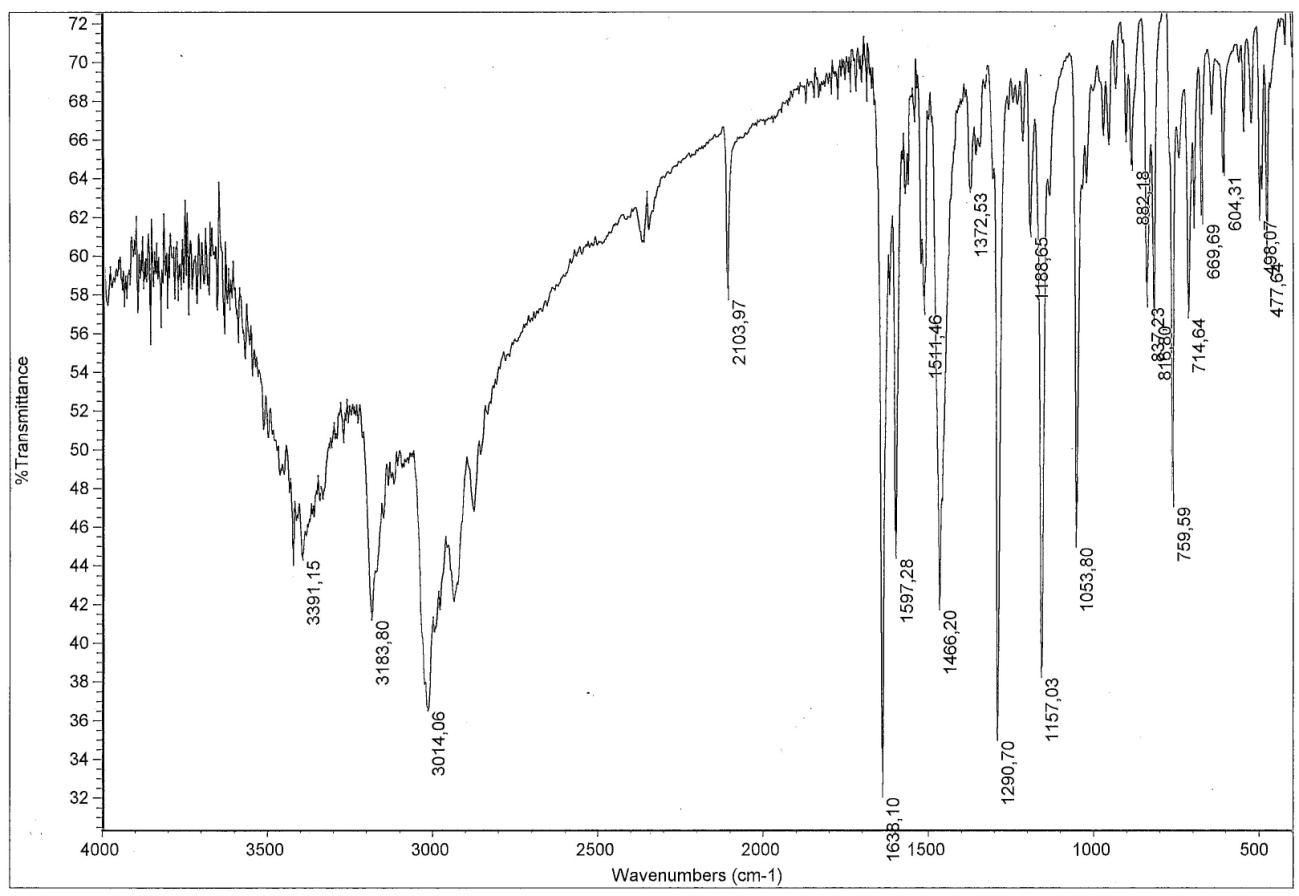
**

**Figure S21**. IR spectrum of **5** in KBr.

**Figure S22**. ^1^H-NMR spectrum of **6** recorded in CDCN_3_ at 400 MHz

**Figure S23**. ^13^C-NMR spectrum of **6** recorded in CDCl_3_ at 100 MHz.


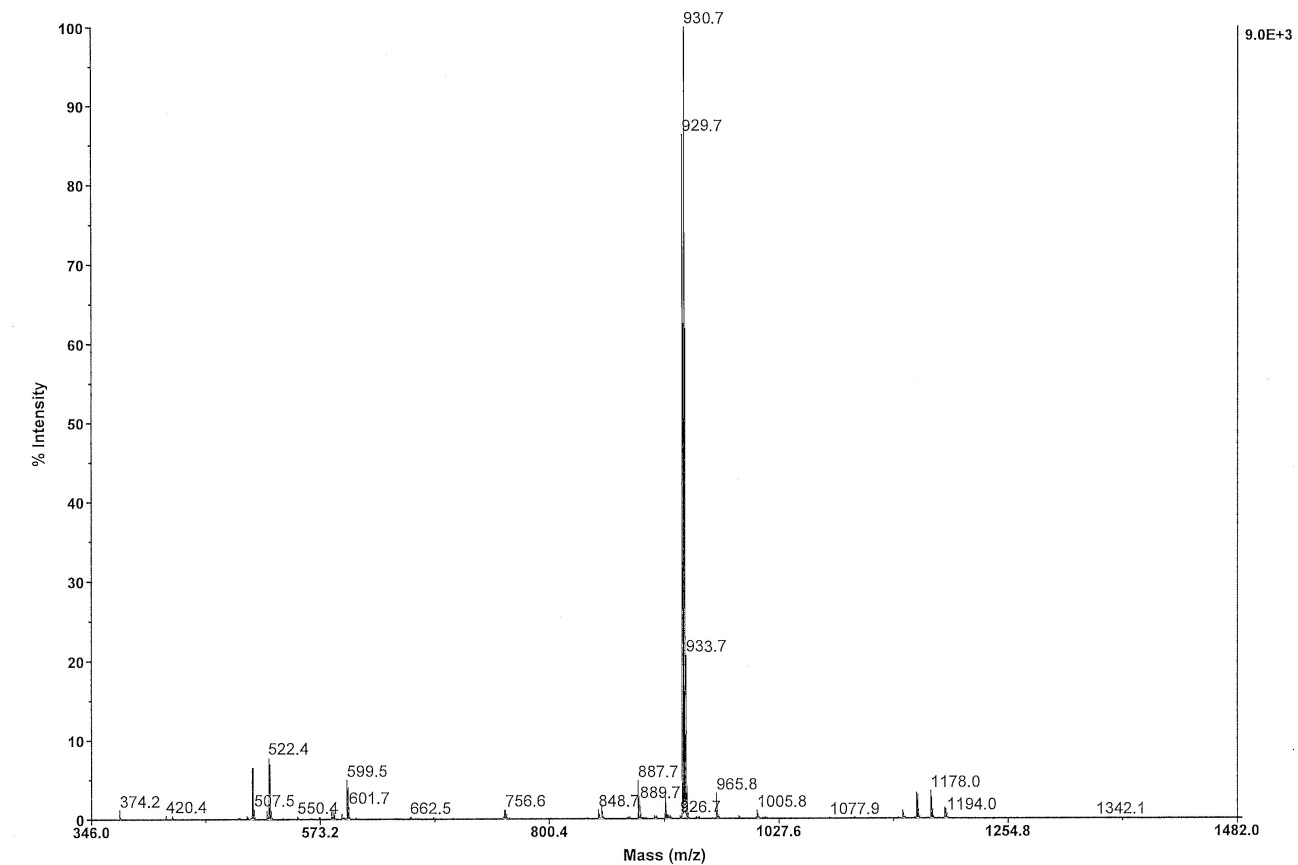


**Figure S24**. MALDI-ToF-MS (m/z) spectrum of **6** with DHB as matrix

**
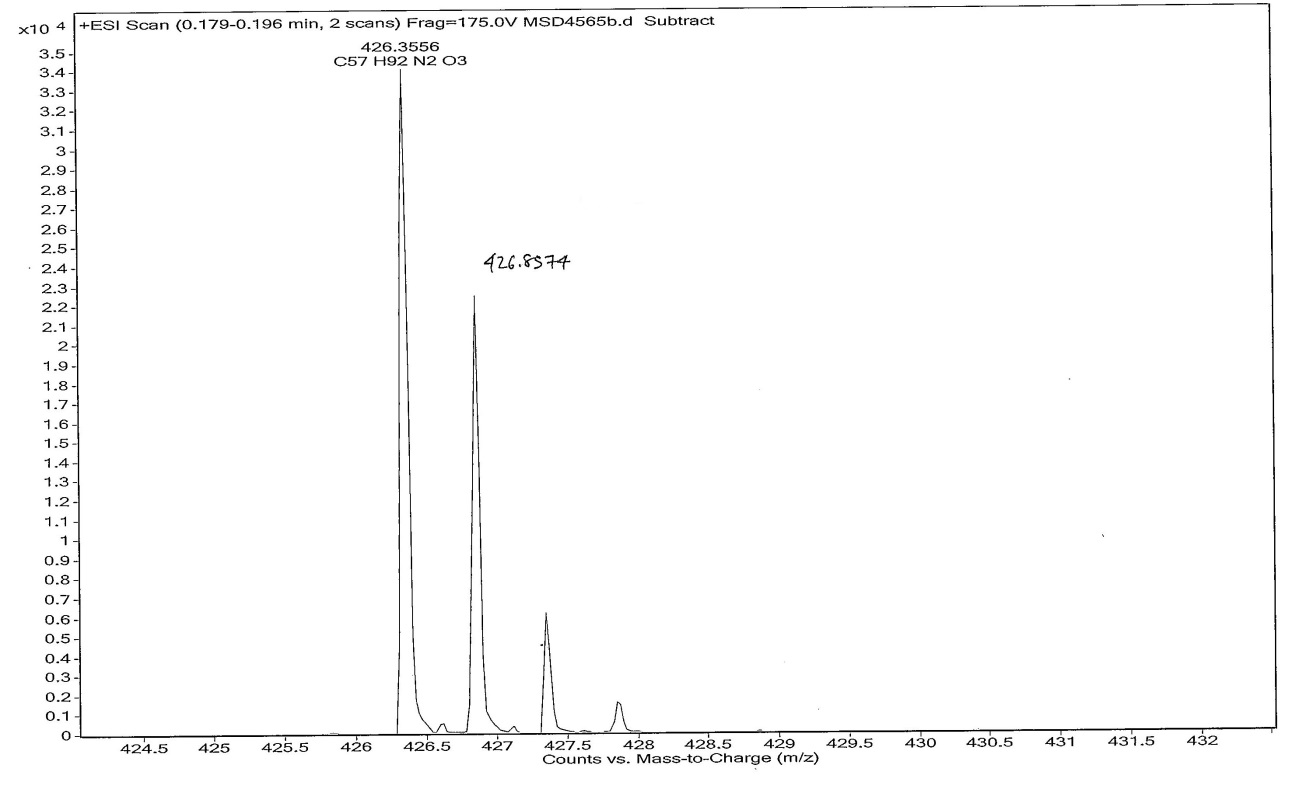
**

**Figure S25.** HMRS-ESI spectrum (m/z) spectrum of **6.**


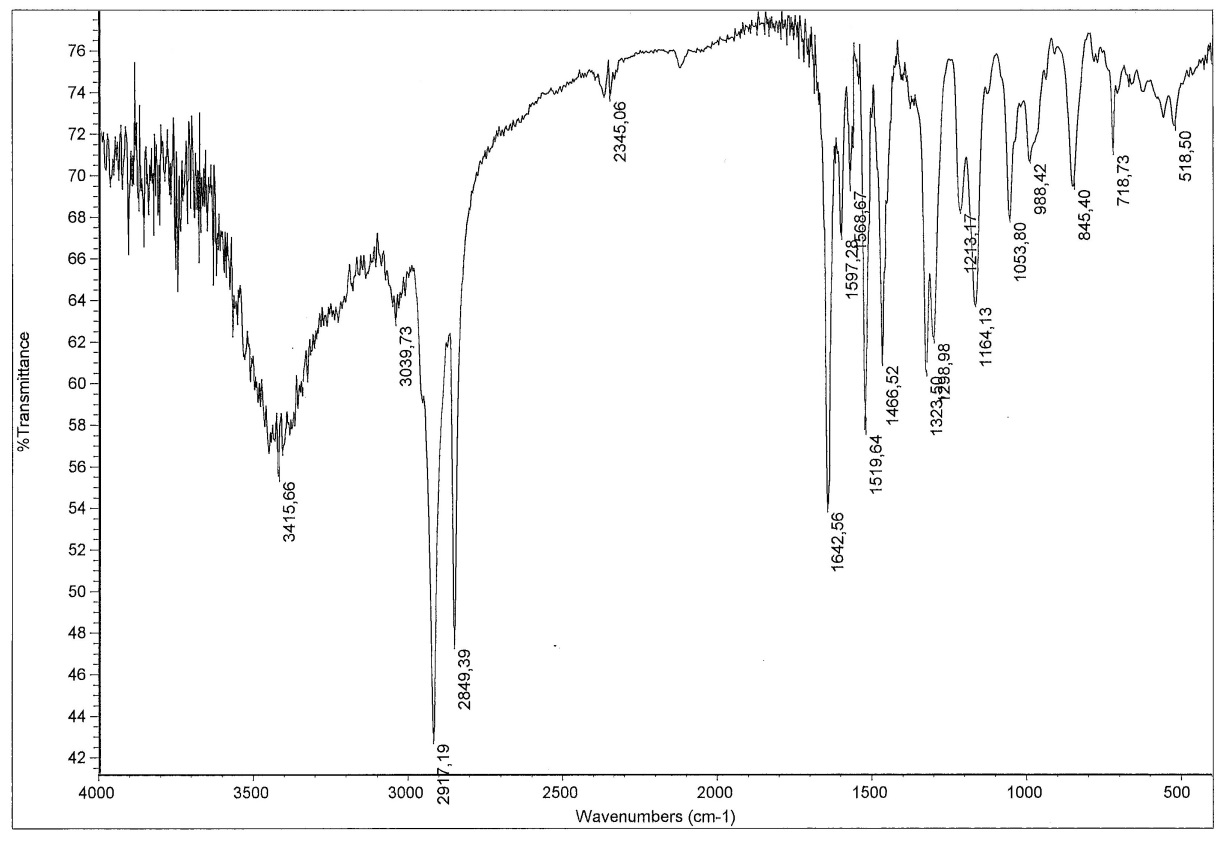


**Figure S26**. IR spectrum of **6** in KBr

**Figure S27**. ^1^H-NMR spectrum of **7** recorded in D_2_O at 400 MHz

**Figure S28**. ^13^C-NMR spectrum of **7** recorded in D_2_O at 100 MHz

**
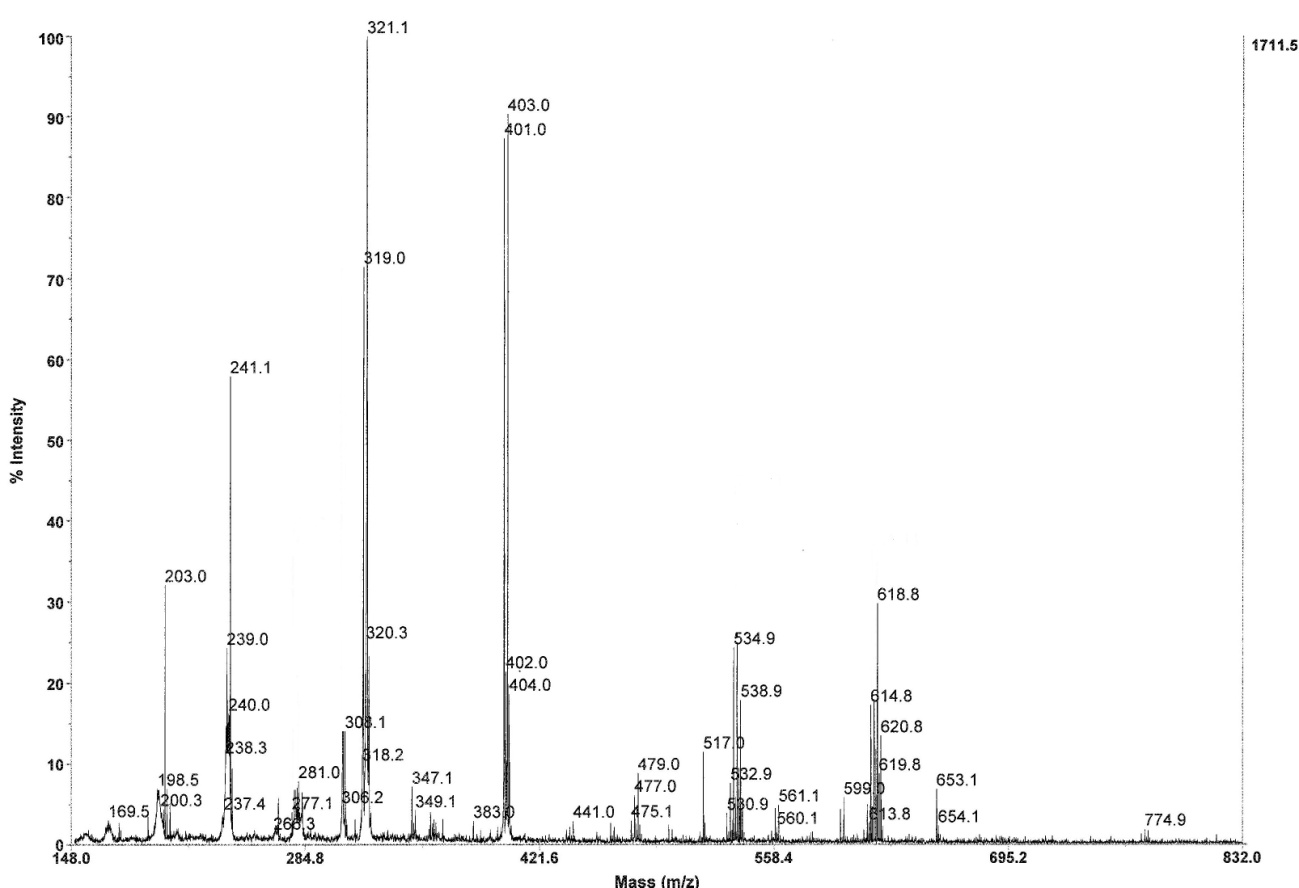
**

**Figure S29**. MALDI-ToF-MS (m/z) spectrum of **7** with DHB as matrix

**
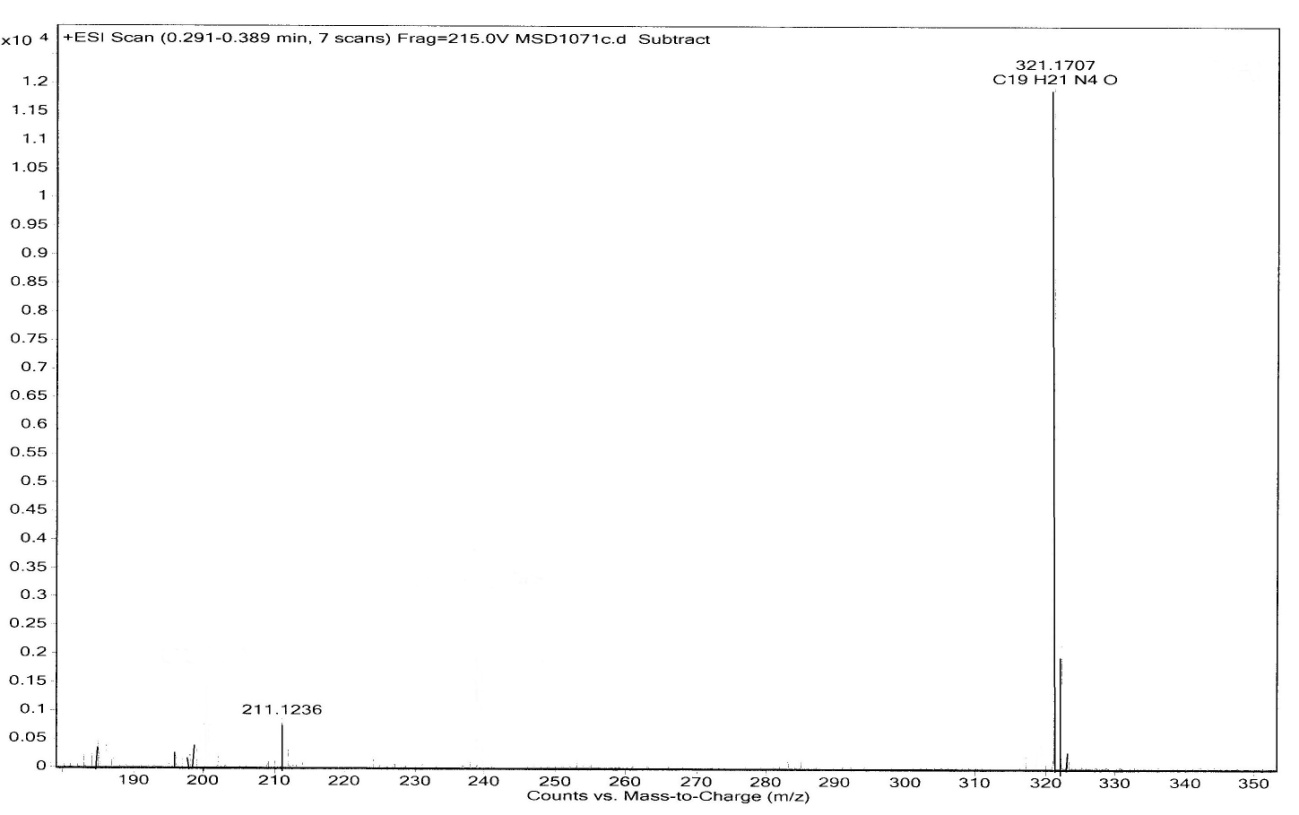
**

**Figure S30**. HMRS-ESI spectrum (m/z) spectrum of **7.**

**
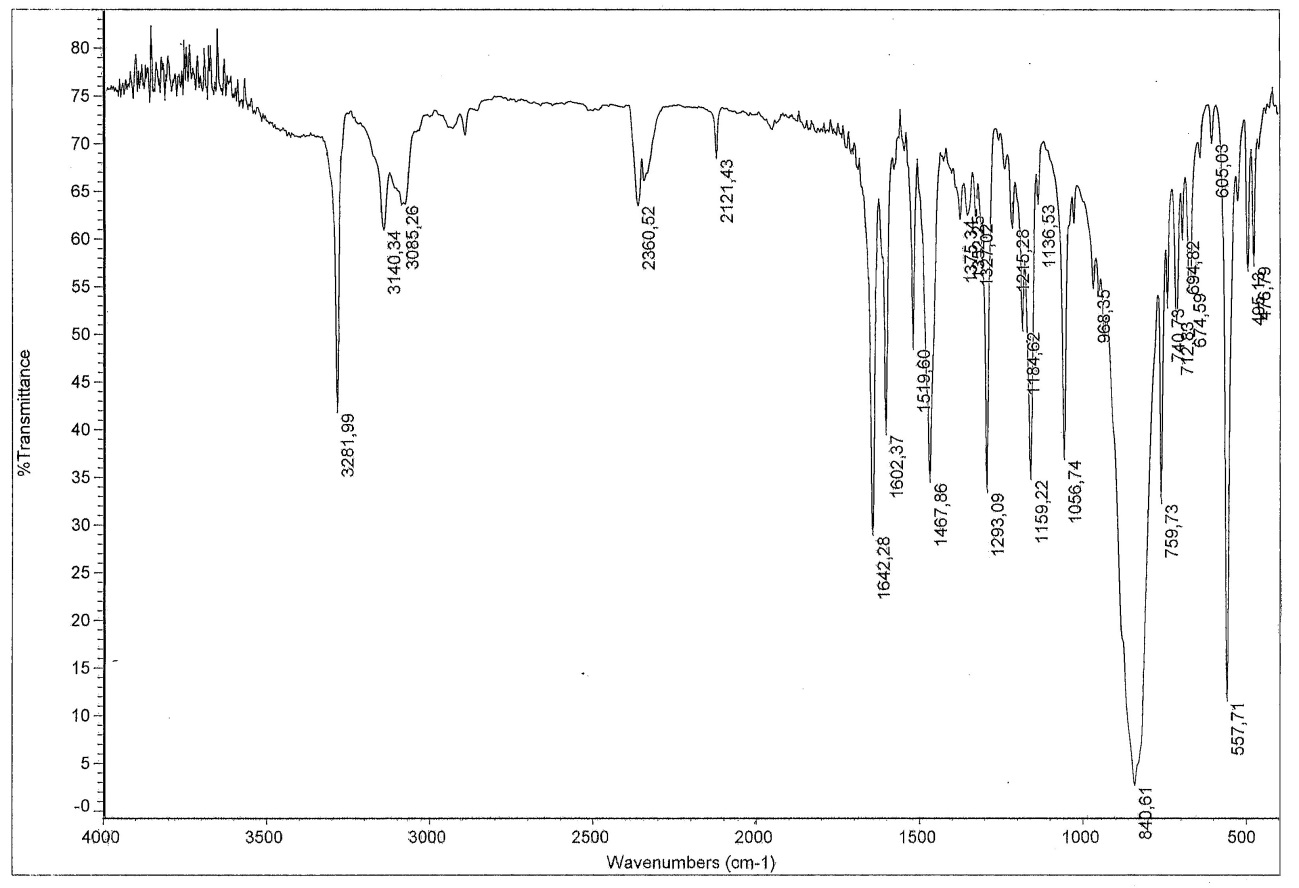
**

**Figure S31**. IR spectrum of **7** in KBr.


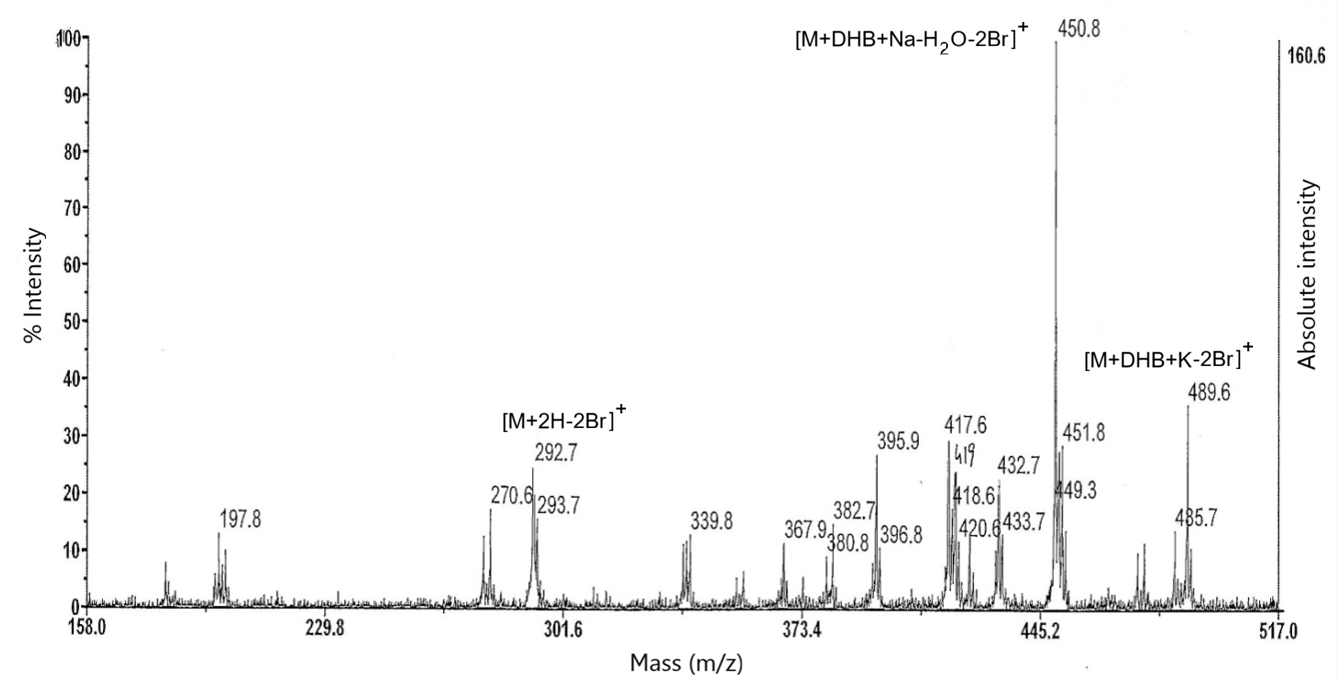


**Figure S32.** MALDI-Tof-MS (m/z) spectrum of PWs functionalized with **1**.


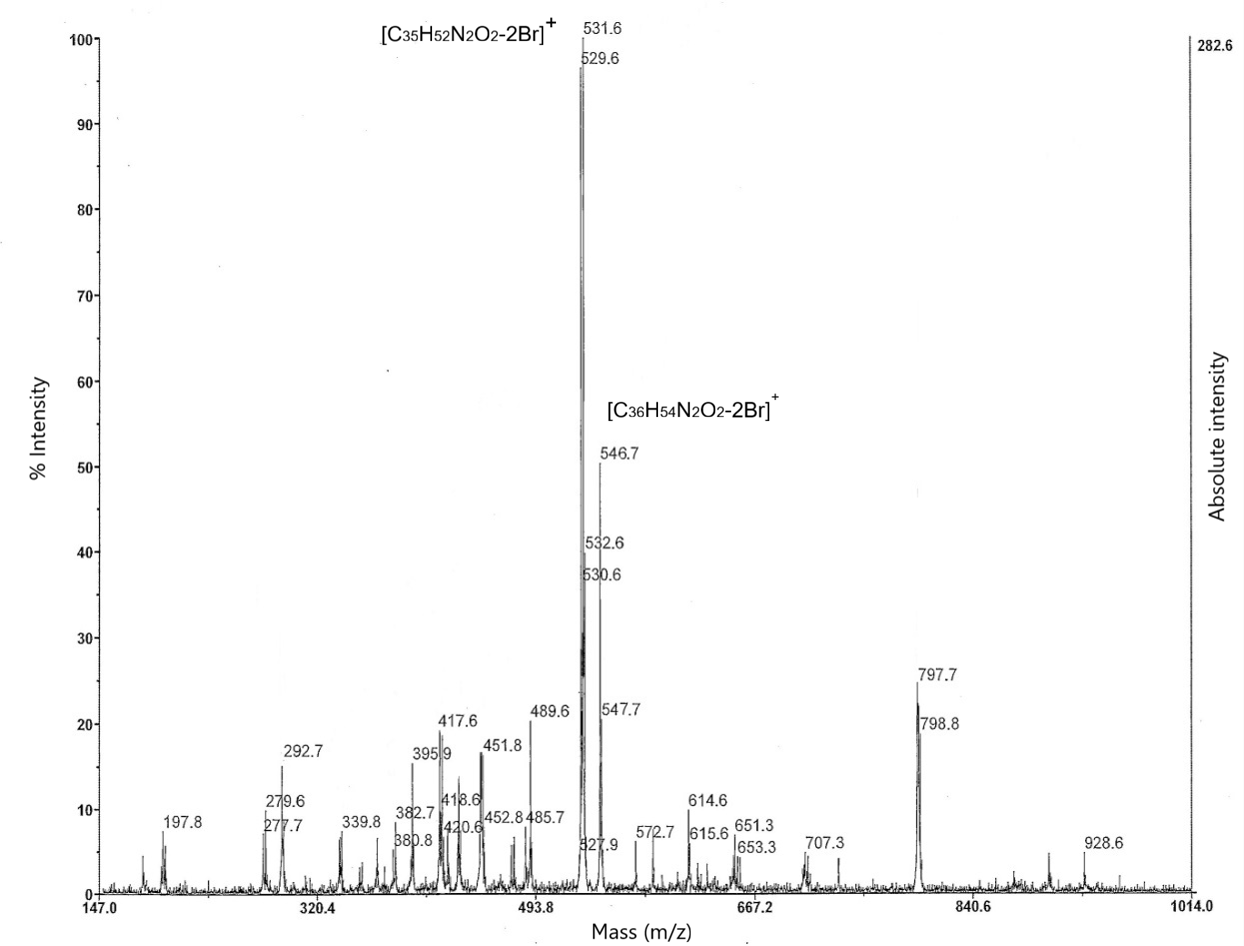


**Figure S33.** MALDI-Tof-MS (m/z) spectrum of PWs functionalized with **2a**.


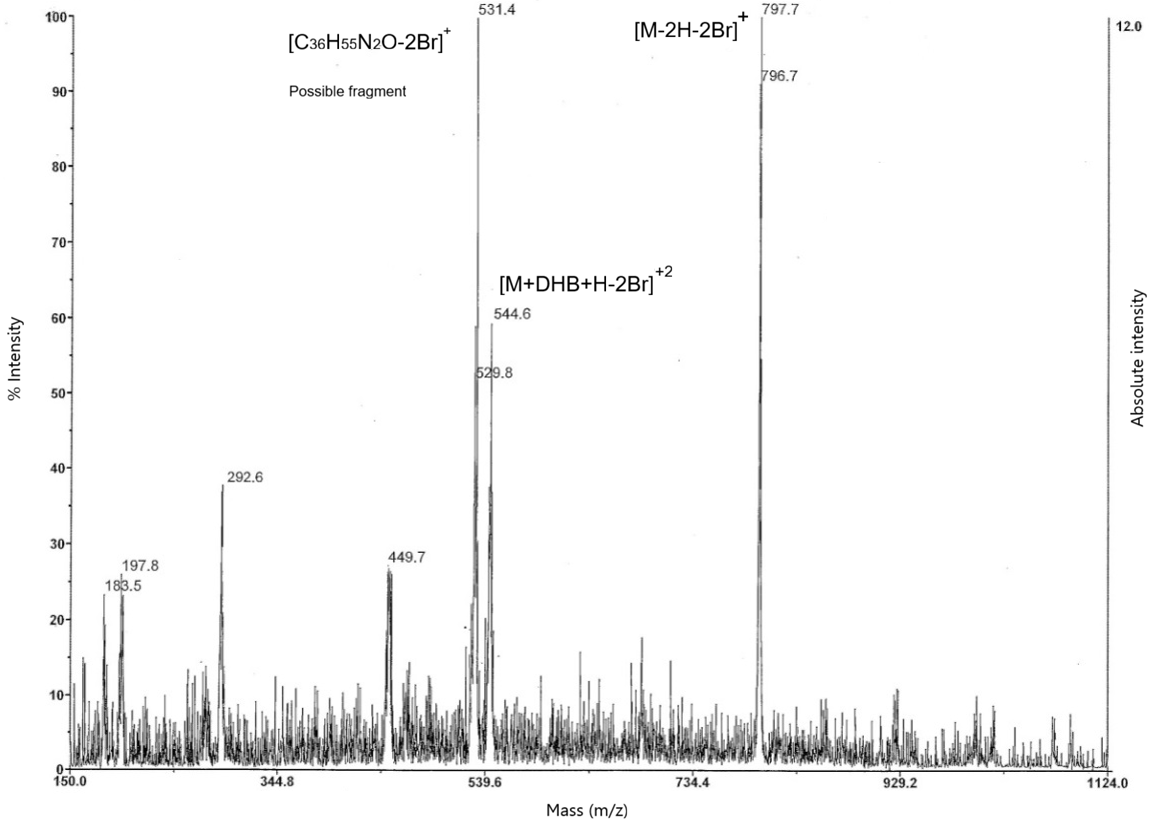


**Figure S34.** MALDI-Tof-MS (m/z) spectrum of PWs functionalized with **2b**.


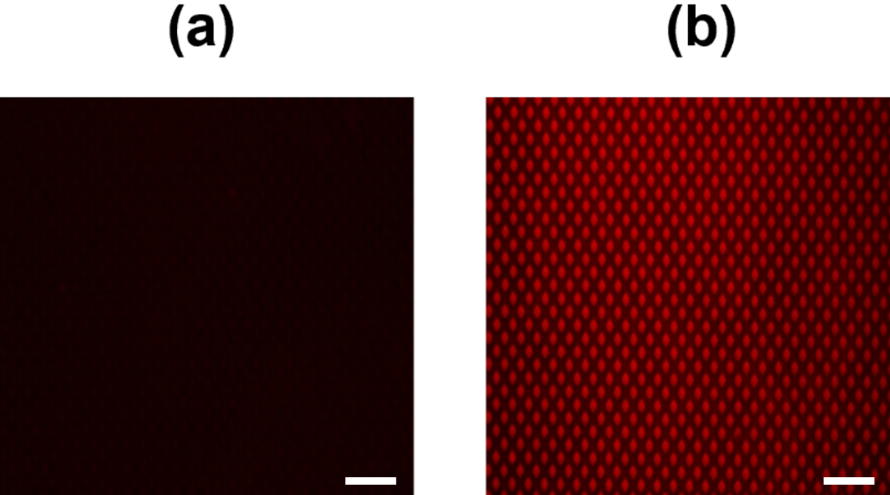


Figure S35. Fluorescence optical microscope image with 200x magnification and two seconds of exposition of PWµCs with 3 × 3 µm polysilicon microchips functionalized. a) after functionalization only with **2a** and e) subsequent immobilization of porphyrin **Na-ZnTCPP**. Scale bars = 20 µm.


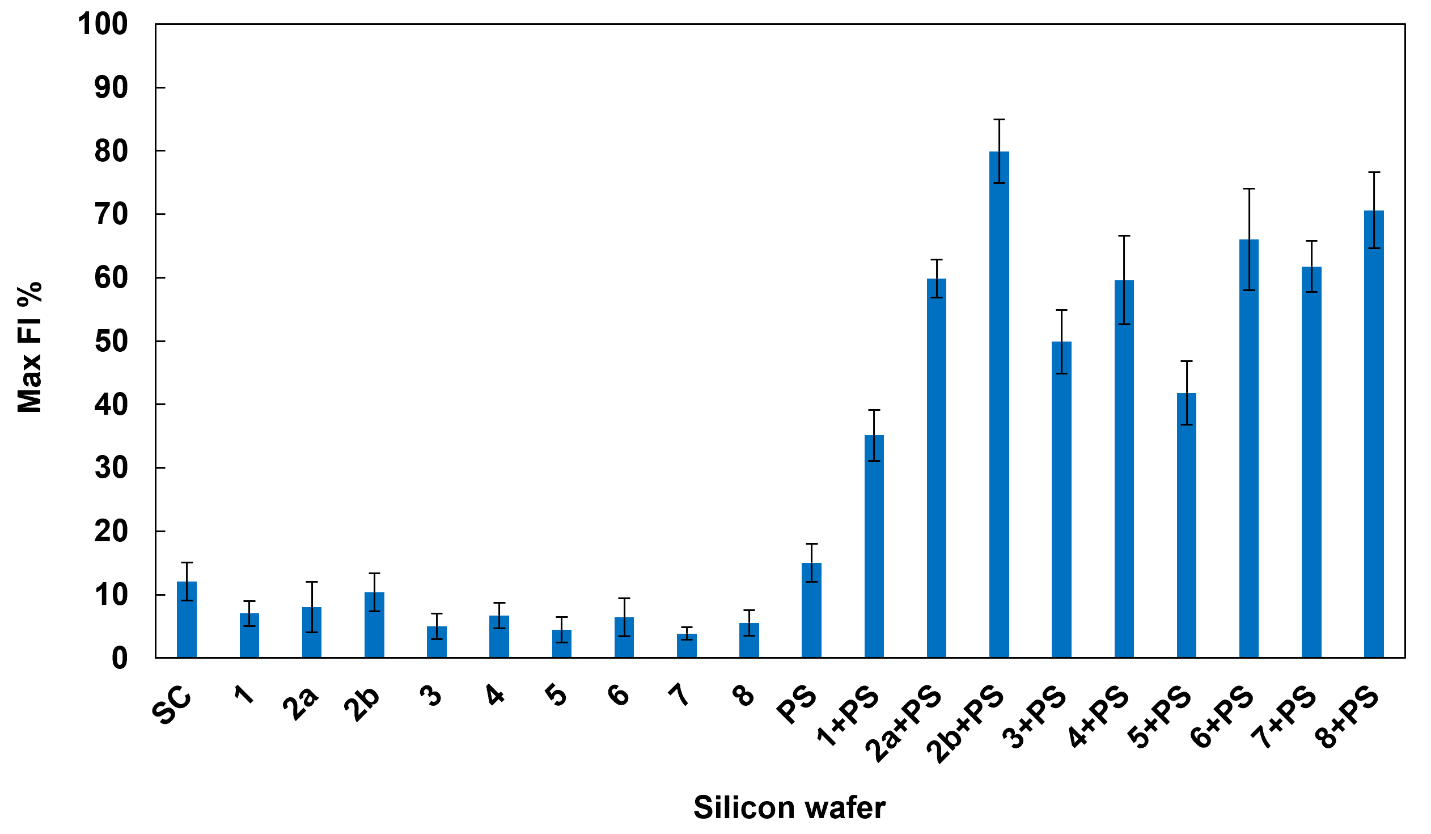


Figure S36. Maximum values of fluorescence intensity (FI) in percentage of: non-functionalized PWµCs, PWµCs functionalized only with bis-pyridinium salts 1-8 or the photosensitizer Na-ZnTCPP (PS), and surfaces functionalized with 1-8 and then with the metalloporphyrin Na-ZnTCPP.


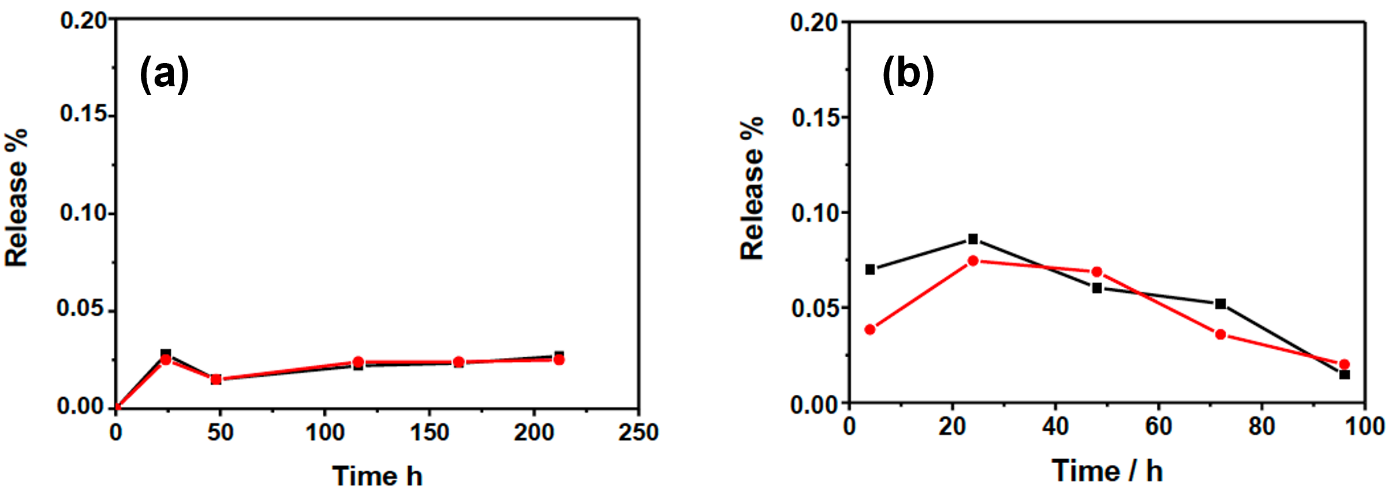


**Figure S37.** Release profile of **Na-ZnTCPP** from PWµCs a) non-covalently with **2** (black line) and **4** (red line), b) covalently with **6** (red line) or with **8** (black line).

The release is expressed as a % of the total amount of porphyrin immobilized on the surface.


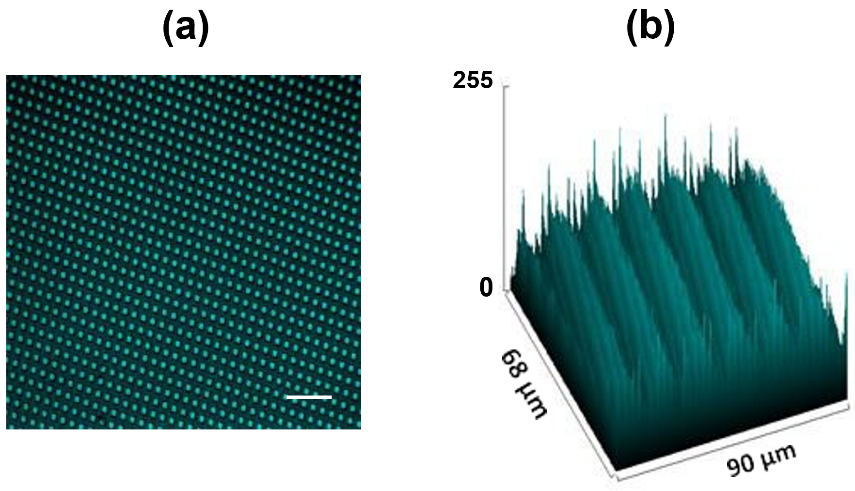


**Figure S38.** Fluorescence optical microscope image of PWµCs with 3 × 3 µm^2^ polysilicon microchips functionalized: a) non-functionalized and b) corresponding 3D images. Scale bar = 20 µm.


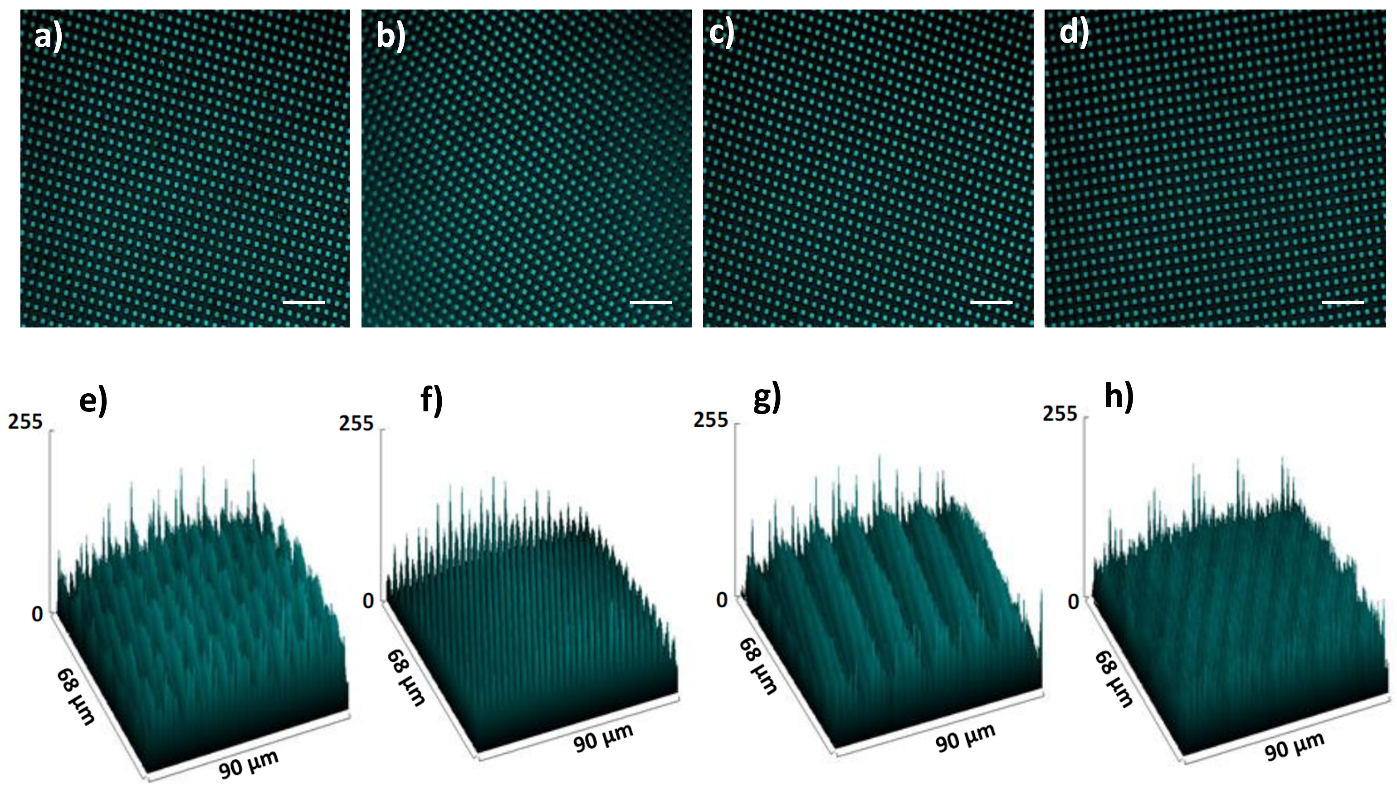


**Figure S39.** Fluorescence optical microscope image of PWµCs with 3 × 3 µm^2^ polysilicon microchips immersed only in solution of the neurotransmitters a) Dop, b) Ser, c) Adr and d) Nor, respectively and e)-h) all their corresponding 3D images. Scale bars = 20 µm.


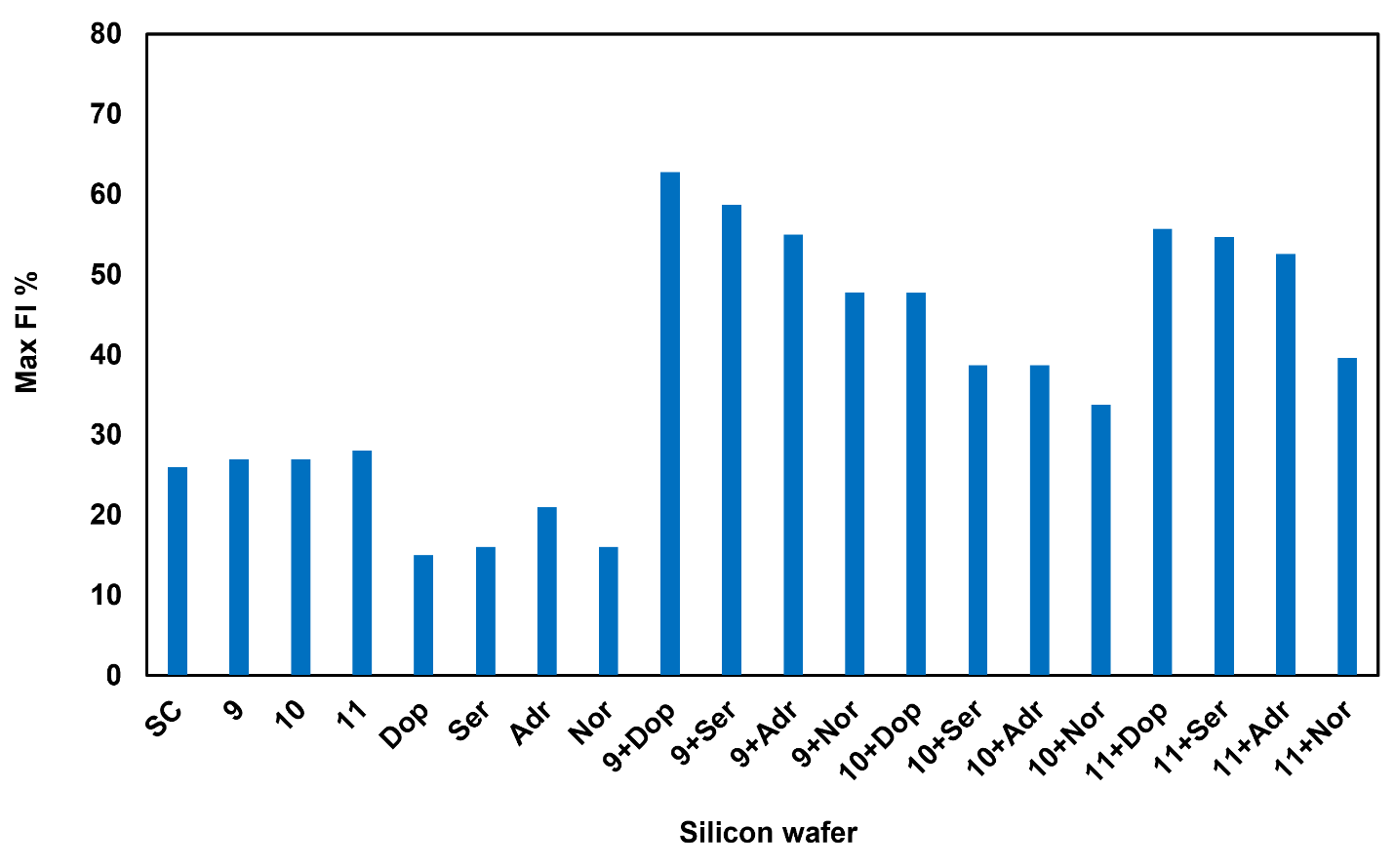


Figure S40. Maximum values of fluorescence intensity (FI) in percentage of non- functionalized PWµCs, PWµCs functionalized only with bis-bipyridinium salts 9, 10 or 11·4PF6 and the neurotransmitters Dop, Ser, Adr or Nor, and surfaces functionalized with 9, 10 or 11 and the subsequent incorporation of Dop, Ser, Adr or Nor as neurotransmitters.


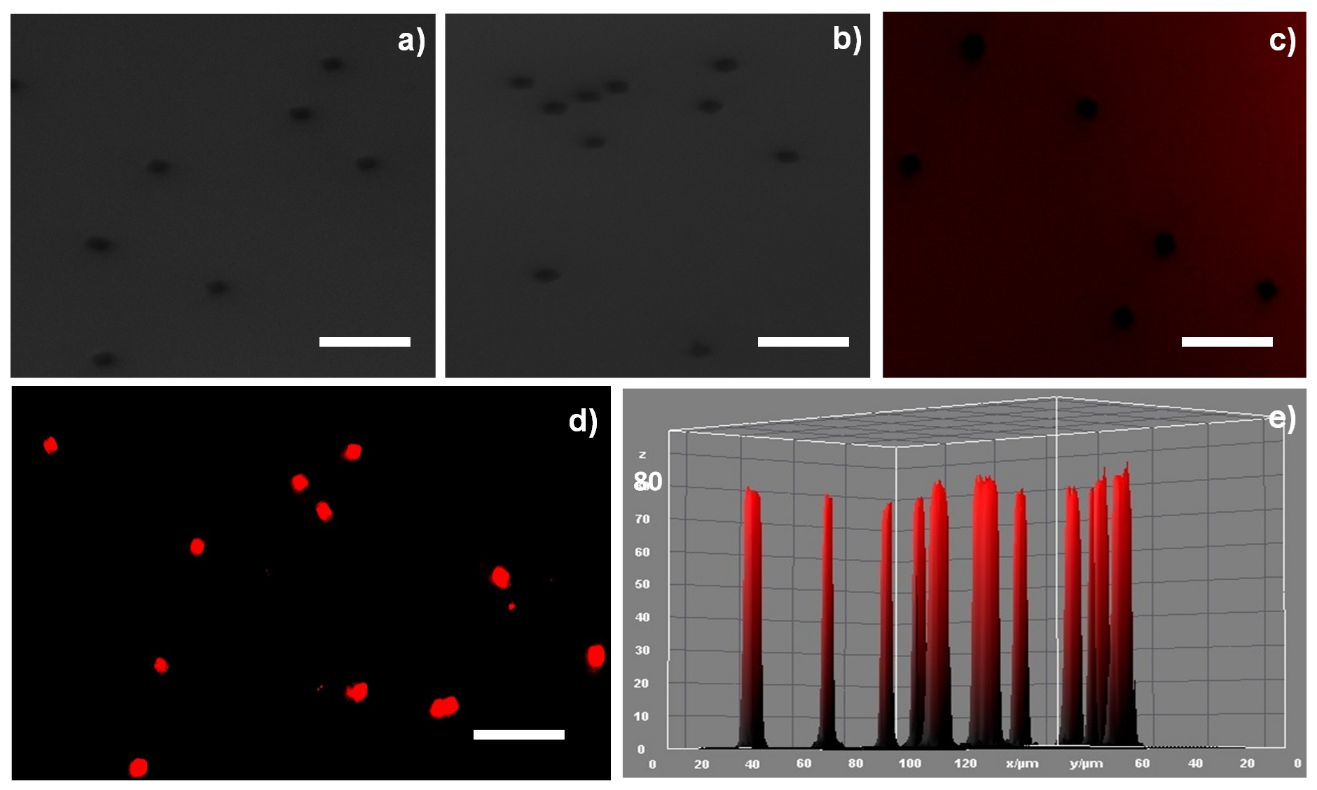


Figure S41. Fluorescence microscopy images of PSµCs with a magnification of 40x and two seconds of exposition of PSµCs: a) control (microparticles non-functionalized) b) after surface activation and functionalization with 2b, c) after surface activation and functionalization with porphyrin Na-ZnTCPP, d) after functionalization with 2b and subsequent immobilization of the porphyrin, and e) their corresponding 3D fluorescence intensity projection. Scale bars = 20 µm.

Figure S42. a) UV-vis absorption spectra of **Na-ZnTCPP** at different concentrations (0.5-10 µM) in water and b) its corresponding calibration curve.


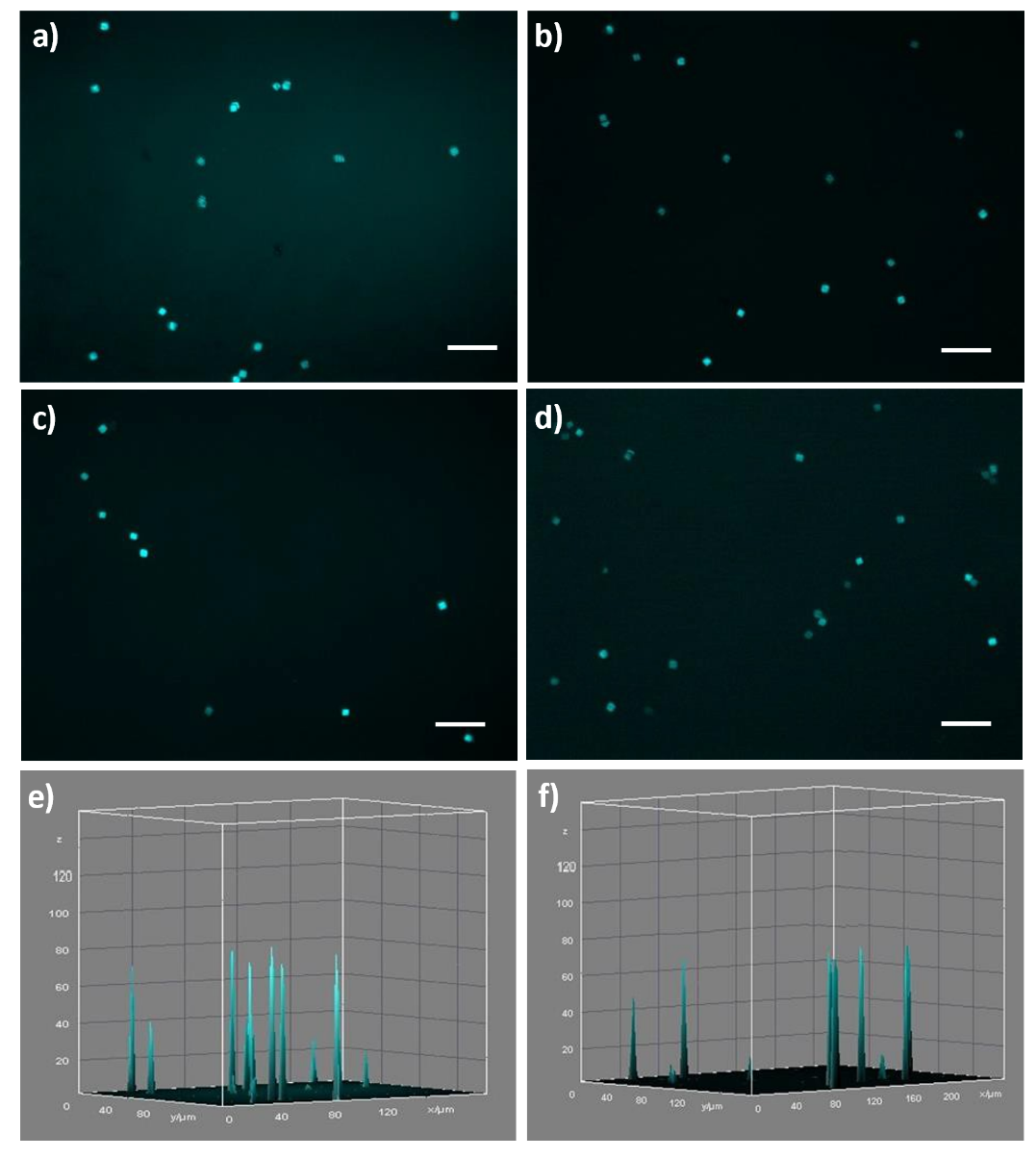


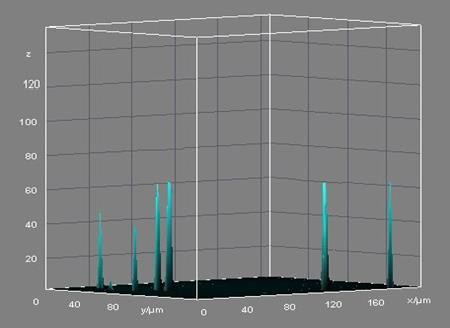


**g)**


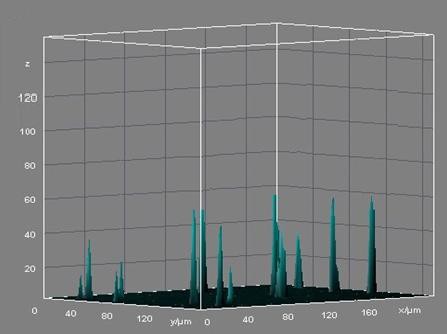


**h)**

**Figure S43.** Fluorescence microscopy images of PSµCs: a) control (microparticles non-functionalized) b) after functionalization only with **9** c) and d) after functionalization with Dop and Ser, respectively and e)-h) their corresponding 3D fluorescence intensity projection. Scale bars = 20 µm.


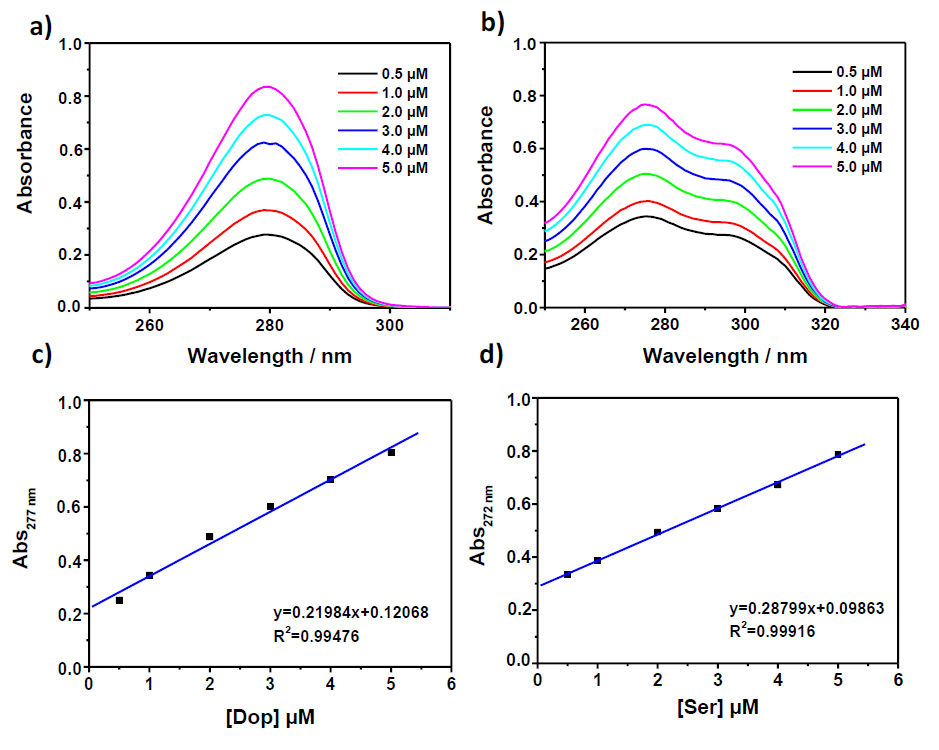


**Figure S44.** a) UV-vis absorption spectra of Dop and b) Ser at different concentrations (0.5-5.0 µM) in water, c) and d) their corresponding calibration curves.

**Table S1**. Concentration of the porphyrin **Na-ZnTCPP** incorporated on PSµCs

| Functionalization of PSµCs | Compound | [Na-ZnTCPP]  µM |
| --- | --- | --- |
| Non-covalent | **2** | 2.3 |
| Non-covalent | **4** | 2.5 |
| Covalent | **6** | 2.2 |
| Covalent | **8** | 2.6 |

Table S2. Covalent vs. non-covalent functionalization of polysilicon microchips with gemini amphiphiles

| **Parameter** | **Covalent functionalization** | **Non-covalent functionalization** | **Key observation from this work** |
| --- | --- | --- | --- |
| Immobilization mechanism | Covalent bond formation via click reaction (e.g., azide–alkyne coupling) | Electrostatic/hydrophobic self-assembly and surface adsorption | Distinct binding modes but similar final surface organization |
| Release / leakage of Na-ZnTCPP over time (>100 h) | ~ 0.080 % | ~ 0.025 % | Ultra-low leakage in both cases; essentially indistinguishable |
|  |  |  |  |
| Fluorescence performance (ZnTCPP signal) | Shrot hydrophobic chain  (40 – 50 %)  Long alkyl chains  (60 – 80 %) | Shrot hydrophobic chain  (40 – 60 %)  Long alkyl chains  (60 – 70 %) | Long alkyl chains (2b, 4, 6, 8) show higher fluorescence intensity |
| Na–ZnTCPP loading (µM; 800,000 PSµCs/mL) | 2.2-2.6 µM | 2.3-2.5 µM | Comparable loading efficiency across both strategies |
|  |  |  |  |
| Synthetic complexity | Multi-step synthesis; amphiphilic salt modification + surface silanization with linker + click reaction, catalysts, purification + Na–ZnTCPP loading | Supramolecular amphiphilic salt immobilization and Na–ZnTCPP loading | Major advantage of non-covalent route |
|  |  |  |  |
| Operational complexity | Higher (strict stoichiometry, reaction time, catalyst control) | Lower (no catalysts) | Non-covalent method significantly simpler |
|  |  |  |  |
| Time requirement | Longer ~ 75 h  (surface activation + silanization + coupling reaction + immobilization) | Shorter ~ 30 h  (surface activation + immobilization) | Operationally faster for non-covalent immobilization |
|  |  |  |  |

**References**

(1) He, C.; Li, L.-W.; He, W.-D.; Jiang, W.-X.; Wu, C. “Click” Long Seesaw-Type A∼∼B∼∼A Chains Together into Huge Defect-Free Hyperbranched Polymer Chains with Uniform Subchains. *Macromolecules* 2011, *44* (16), 6233–6236. https://doi.org/10.1021/ma2013783.
